# The Structure of the RNA-Dependent RNA Polymerase of a Permutotetravirus Suggests a Link between Primer-Dependent and Primer-Independent Polymerases

**DOI:** 10.1371/journal.ppat.1005265

**Published:** 2015-12-01

**Authors:** Diego S. Ferrero, Mònica Buxaderas, José F. Rodríguez, Núria Verdaguer

**Affiliations:** 1 Institut de Biologia Molecular de Barcelona, CSIC, Parc Científic de Barcelona, Barcelona, Spain; 2 Centro Nacional de Biotecnología, CSIC, Madrid, Spain; Institut Pasteur, FRANCE

## Abstract

Thosea asigna virus (TaV), an insect virus belonging to the *Permutatetraviridae* family, has a positive-sense single-stranded RNA (ssRNA) genome with two overlapping open reading frames, encoding for the replicase and capsid proteins. The particular TaV replicase includes a structurally unique RNA-dependent RNA polymerase (RdRP) with a sequence permutation in the palm sub-domain, where the active site is anchored. This non-canonical arrangement of the RdRP palm is also found in double-stranded RNA viruses of the *Birnaviridae* family. Both virus families also share a conserved VPg sequence motif at the polymerase N-terminus which in birnaviruses appears to be used to covalently link a fraction of the replicase molecules to the 5’-end of the genomic segments. Birnavirus VPgs are presumed to be used as primers for replication initiation. Here we have solved the crystal structure of the TaV RdRP, the first non-canonical RdRP of a ssRNA virus, in its apo- form and bound to different substrates. The enzyme arranges as a stable dimer maintained by mutual interactions between the active site cleft of one molecule and the flexible N-terminal tail of the symmetrically related RdRP. The latter, partially mimicking the RNA template backbone, is involved in regulating the polymerization activity. As expected from previous sequence-based bioinformatics predictions, the overall architecture of the TaV enzyme shows important resemblances with birnavirus polymerases. In addition, structural comparisons and biochemical analyses reveal unexpected similarities between the TaV RdRP and those of Flaviviruses. In particular, a long loop protruding from the thumb domain towards the central enzyme cavity appears to act as a platform for *de novo* initiation of RNA replication. Our findings strongly suggest an unexpected evolutionary relationship between the RdRPs encoded by these distant ssRNA virus groups.

## Author Summary

RNA dependent RNA polymerases (RdRPs) are the catalytic components of the RNA replication and transcription machineries, and thus central players in the life cycle of RNA viruses. The in-depth understanding of both the structure and regulation of viral RdRPs displaying different replication-transcription strategies might provide essential clues for an effective control of virus propagation. The characterization of the first non-canonical RdRP of a positive-stranded RNA virus, the permutotetravirus *Thosea asigna* virus, has unveiled two essential elements controlling polymerization activity: (i) the protein N-terminus that invades the central cleft of the neighboring RdRP molecule, thus stabilizing a dimeric form of the enzyme with partially occluded template binding channels; and (ii) a long loop protruding towards the catalytic cavity which harbors the binding site of incoming nucleotides, thus providing a platform for *de novo* replication initiation. The close structural and functional resemblance between this enzyme and flaviviral RdRPs strongly suggests the existence of an unexpected evolutionary link between these two distant virus groups.

## Introduction

RNA viruses strictly depend upon their RNA-dependent polymerases (RdRPs) for genome transcription and replication. Detailed structural and functional knowledge of RdRPs using different replication-transcription strategies may provide essential clues for the control of virus propagation. Although RdRPs share limited sequence similarities, their three-dimensional structures and mechanisms of action are closely related. All RdRPs have a closed right hand-like shape encircling seven motifs, A to G, containing highly conserved amino acids that are essential for polymerase function [1,2]. The four so-called palm motifs, arranged in the order A, B, C and D, are the most conserved feature of viral RdRPs, with motifs A and C containing the catalytic aspartic acid residues [1]. Exceptions to this design have been reported in members of the *Birnaviridae* and *Permutotetraviridae* families, harboring double-stranded (ds) and positive (+) single-stranded (ss) RNA genomes, respectively. In these enzymes, motif C is located upstream of motif A forming a non-canonical C-A-B arrangement with a unique connectivity of the major structural elements of the active site [3,4].

Non-canonical palm connectivity has also been described in the RdRP encoded by Grapevine virus Q (GVQ) [5], an alpha-like plant tymovirus. However, bioinformatics analyses suggest that whereas the permuted RdRPs from birna- and permutotetraviruses share a monophyletic origin that of GVQ evolved independently [5].

Besides the permuted connectivity of the palm subdomain, birna- and permutatretravirus replicases also share a conserved N-terminal region, including a VPg sequence motif (Y/FXXGS/TXXGXXXRL) that in birnaviruses seems to be used to covalently link a fraction of the replicase molecules to the 5′-end of the genome segments. Birnavirus VPg molecules are likely used as primers for replication [6–8]. Furthermore, it has been hypothesized that the putative VPg signal of permutatetraviruses would be also for RNA synthesis priming [3,4].

The X-ray structures of permuted RdRPs from two dsRNA viruses, i.e. infectious bursal disease virus (IBDV) and infectious pancreatic necrosis virus (IPNV), belonging to the *Birnaviridae* family, have been reported. Despite their non-canonical connectivity, the overall architecture of their catalytic sites is akin to those of canonical RdRPs [9–11]. Indeed, the structural similarities of birnavirus RdRPs to their picorna- and calicivirus counterparts conveyed key evidence supporting the existence of an evolutionary link connecting dsRNA birnaviruses and +ssRNA viruses [9]. In addition, a structure-based mutational analysis on the IPNV RdRP revealed that an N-terminal serine residue is required for the formation of covalent RdRP-RNA complexes [11].

Despite the obvious structural and functional interest as well as its critical importance for the understanding of evolutionary relationships between dsRNA and +ssRNA viruses sharing permuted RdRP palms, information about non-canonical +ssRNA RdRPs was missing. In this report, we describe the structural and functional characterization of the RdRP domain of the permutotetravirus *Thosea asigna* virus (TaV), an insect virus which infects larvae of *Setothosea asigna* (*Lepidoptera*), the major defoliating pest of oil and coconut palms in Southeast Asia. Progress in the molecular characterization of TaV, as well as other members of this family, has been hampered by the difficulty in growing them in tissue culture [12]. Our results include the first crystal structures of the TaV enzyme in its apo form (2.15 Å resolution), bound to CTP and to GTP (2.25 Å and 2.3 Å resolution, respectively), and to a short ssRNA template in the presence of an incoming ATP (3.5 Å).

Surprisingly, the TaV RdRP structures closely resemble those of polymerases encoded by flaviviruses (a family that includes important human pathogens as dengue [DV], West Nile [WNV] or hepatitis C [HCV] viruses), exhibiting a number of peculiarities typically found in enzymes using *de novo* replication initiation mechanisms. Of particular importance is the presence of a long loop protruding from the thumb subdomain that is the binding site for the incoming rNTP as evidenced in the structure of the RdRP-ssRNA-ATP ternary complex. Additionally, *in vitro* polymerization assays show that the TaV enzyme is active in a primer-independent reaction, thus confirming the existence of a functional relationship with flaviviral RdRPs.

## Results

### Characterization of TaV ORF-derived constructs

**Fig 1:**
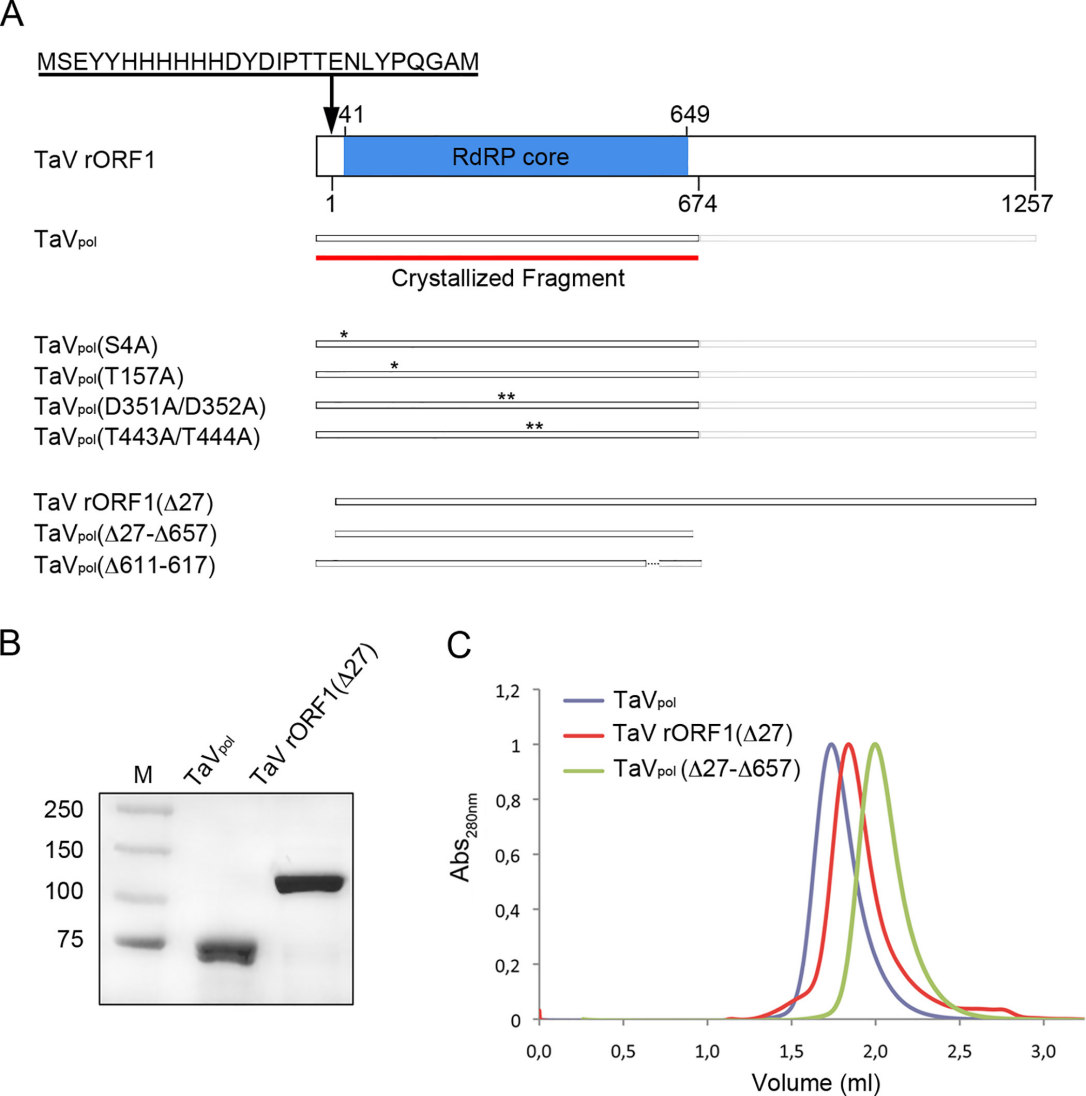
Production of TaV ORF1-derived constructs. (A) The cartoon schematically depicts protein constructs used in this report. The red line indicates the crystallized fragment containing the RdRP domain (TaV_pol_). The polyhistidine tag, located at the N-terminus of each recombinant protein, is indicated at the top. Positions of substituted amino acids in mutant protein versions are indicated as “*”. (B) Comassie blue-stained SDS-PAGE corresponding to purified TaV_pol_ and TaV rORF1(Δ27) constructs. Lane M corresponds to molecular mass markers (kDa). (C) Analytic chromatography on a calibrated Superdex 200 5/150 column of purified TaV_pol_ (blue), TaV rORF1(Δ27) (red), and TaV_pol_(Δ27-Δ657) (green).

The full-length TaV ORF1 (140 kDa) fused to an N-terminal tail containing an hexa-histidine Tag (TaV rORF1; Fig 1A) was expressed in Hi5 insect cells infected with a recombinant baculovirus, rBV-TaV ORF1. After expression, the recombinant protein was rapidly cleaved releasing a 75 kDa protein fragment (Fig 1B) [13]. Mass spectrometry (MALDI-TOF/TOF) showed that this polypeptide harbors the first 674 residues of the recombinant protein, including the whole RdRP domain (TaV_pol_ from here on; Fig 1A). The final purification step, size exclusion chromatography, showed that TaV_pol_ is a dimer in solution (Fig 1C). This construct was used for both crystallographic analyses and functional characterization of the RdRP activity. In addition, other protein constructs, harboring mutations at the active site motifs C TaV_pol_(D351A/D352A) and B TaV_pol_(T443A/T444A) and at the two putative nucleotidylation sites, TaV_pol_(S4A) and TaV_pol_(T157A), or deletions at the N- and/or C-terminus of the protein, TaV rORF1(Δ27), TaV_pol_(Δ27-Δ657) and TaV_pol_(Δ611–617), were also expressed and purified (Fig 1A). It is important to note that the TaV rORF1(Δ27) mutant gene resulted in a protein variant that does not undergo a significant proteolytic degradation in insect cells, thus allowing the purification of the whole polypeptide (Fig 1B). Strikingly, this protein appears to be a monomer in solution as determined by analytical size exclusion chromatography (Fig 1C). The truncated version TaV_pol_(Δ27-Δ657) is also a monomer (Fig 1C). A small amount of purified full-length TaV rORF1 was obtained and used in subsequent activity assays.

### *In vitro* polymerase activity of TaV rORF1 and TaV_pol_

**Fig 2:**
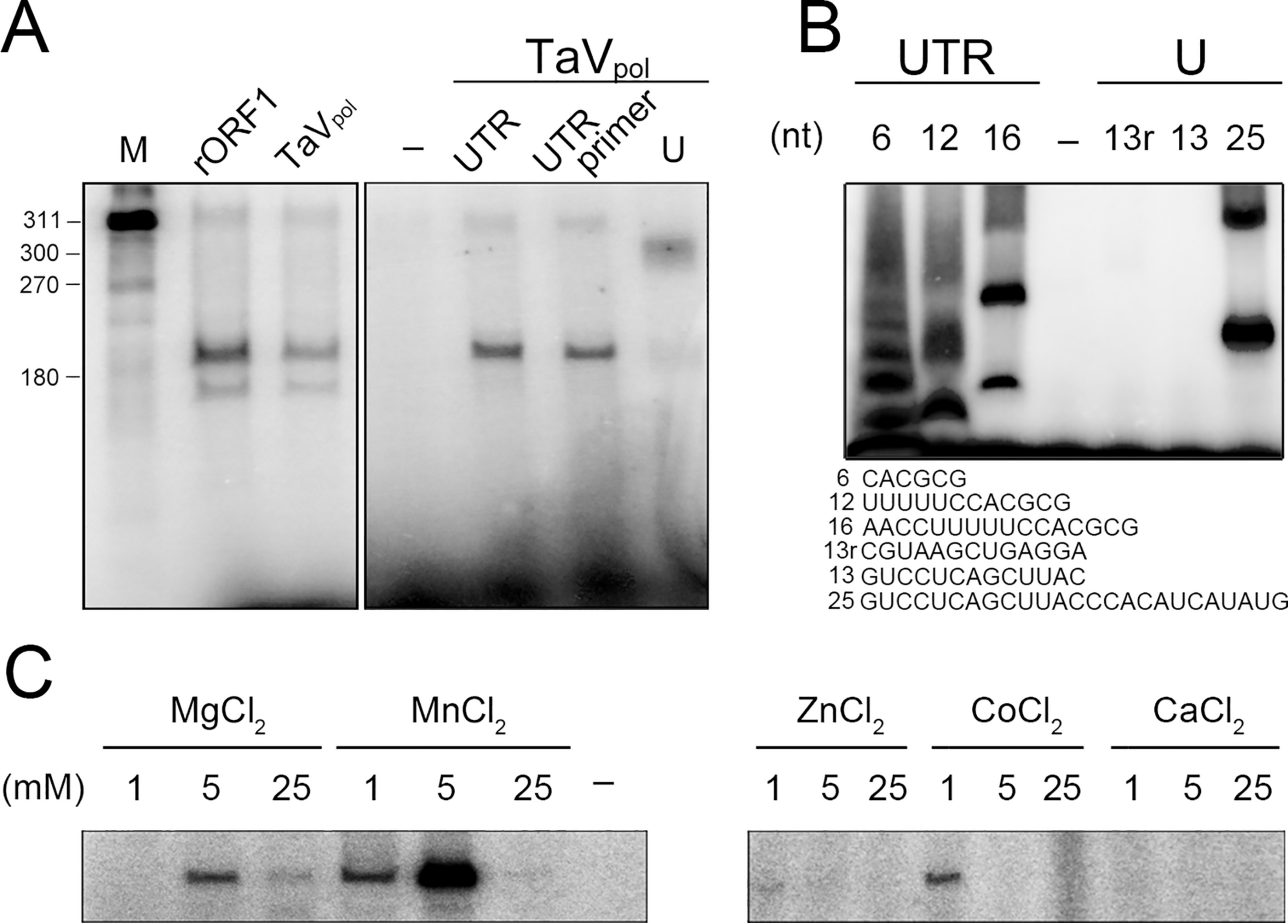
*In vitro* polymerase activity of TaV rORF1 and TaV_pol_. Representative autoradiograms of *in vitro* TaV RdRP activity assays analyzed in 7% acrylamide TBE gels. (A) Left panel, first lane, ssRNA template labeled with α-^32^P GTP; second and third lines, RdRP activities of TaV rORF1 and TaV_pol_, respectively, using the previously unlabelled 311-nts long ssRNA template corresponding to the TaV 3’-UTR. Right panel, polymerization activity of TaV_pol_ performed in: i) absence of template (first lane), ii) presence of the TaV 3’-UTR template (second lane), iii) presence of the TaV 3’-UTR hybridized to a short oligonucleotide primer complementary to an internal template sequence (third lane) and, iv) presence of a non-related viral template (the 3’-UTR of the SJNNV nodavirus; fourth lane). (B) Polymerization activity of TaV_pol_ on short ssRNA templates. Oligonucleotides of 6-, 12- and 16-nts in length (left) derive from the TaV 3’-UTR and the 13- and 25-nts in length (right) contain TaV unrelated sequences. The negative control (-) was performed in absence of RNA. (C) Different ions (Mg^2+^, Mn^2+^, Zn^2+^, Co^2+^, Ca^2+^) and concentrations ranging from 1 to 25 mM were employed in polymerization experiments performed under otherwise optimal conditions (see Methods and S1 Fig). A negative control (-) was performed using a reaction mixture lacking metal ions.

The *in vitro* RNA synthesis activities of the full length TaV rORF1 and its RdRP domain TaV_pol_ were first analyzed using a ssRNA template derived from the 3’ untranslated region (UTR) of the TaV genome [4], demonstrating that both constructs are able to synthesize dsRNA from a ssRNA template in the absence of primer, in a reaction dependent of Mg^2+^ as catalytic ion (Fig 2). The RdRP activity of TaV_pol_ was also tested in the presence of a short RNA primer (8-nts) complementary to an internal sequence of the TaV 3’-UTR, showing equivalent levels of RNA synthesis (Fig 2A). In addition, the use of ssRNA templates of totally heterologous sequences (as the 3’-UTR of a nodavirus genome; Fig 2A) indicates that, at least *in vitro*, the TaV enzyme does not require specific template sequences or secondary structures for polymerization. RNA polymerization activity was also observed on short RNA templates (from 6 to 25 nucleotides) harboring either unrelated or TaV 3’-UTR-derived sequences (Fig 2B). These data illustrate that, although TaV_pol_ is able to carry out *de novo* RNA synthesis on small non-specific templates, the presence of a guanine at the 3’-end of the template seems to be necessary to initiate the reaction.

Like the rest of the well-known polymerases, the RdRP activity of TaV_pol_ is strictly dependent on metal ions as Mg^2+^ and Mn^2+^ (Fig 1C). As described before [14–16], the cofactor Mn^+2^ strongly enhances RNA synthesis. In contrast to what was observed for the non-canonical IBDV RdRP [17], only residual activity was observed in presence of 1 mM Co^2+^ (Fig 1C). Furthermore, the replacement of either Mg^2+^ or Mn^2+^ by other divalent cations, i.e. Ca^2+^ or Zn^2+^, exerts a clear inhibitory effect on RNA synthesis (Fig 1C). The polymerization kinetics analysis performed under optimal conditions for this enzyme (1.3 mM RdRP in 50 mM MES pH 6, 150 mM NaCl and 5 mM MgCl_2_ at 35°C; S1 Fig) shows a sigmoid profile with an initial step of low RNA synthesis and an end state of saturation (S2 Fig).

### The structure of TaV_pol_

**Fig 3:**
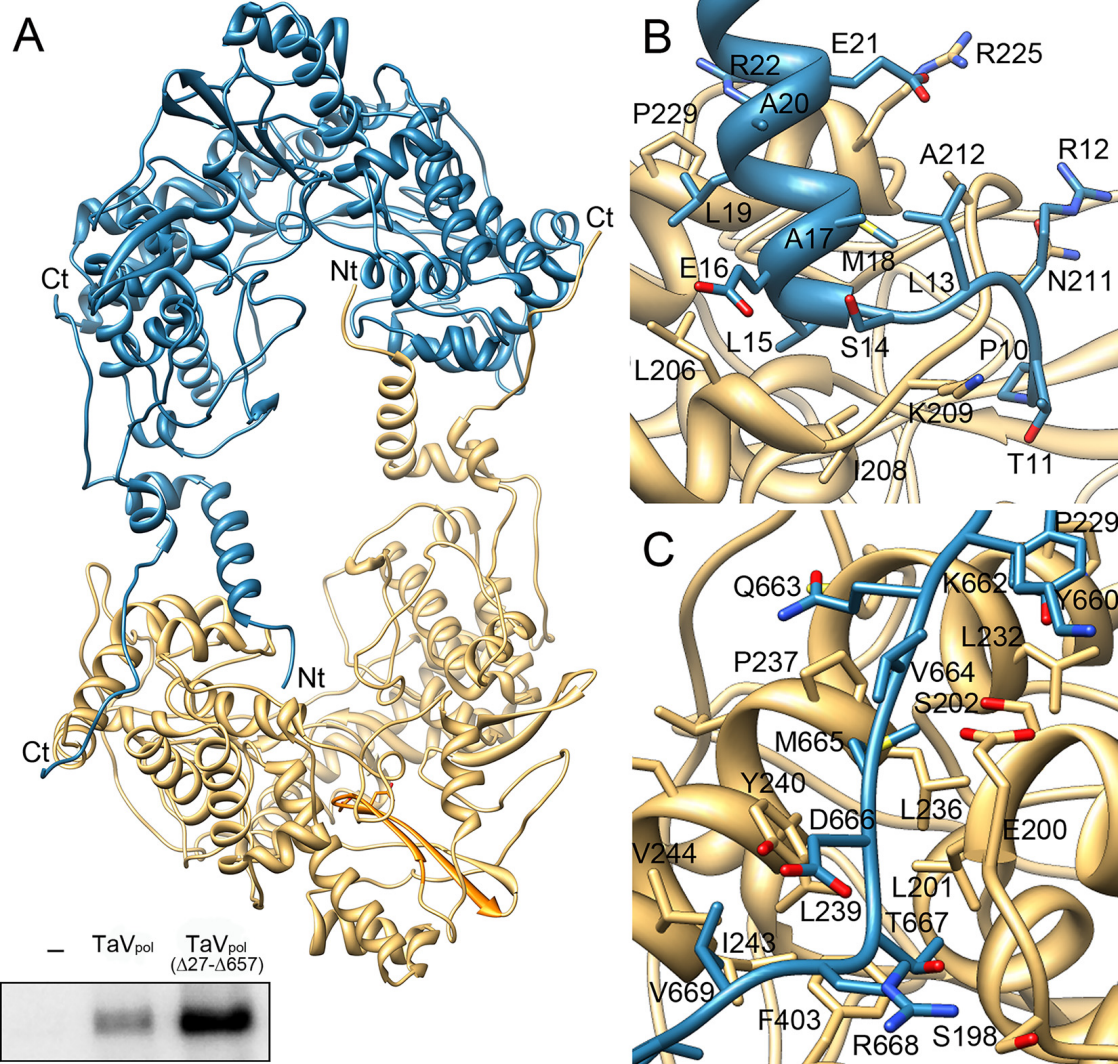
Structure of the TaV_pol_ dimer. (A) The two molecules of the crystal asymmetric unit are shown as ribbons in blue and wheat, respectively. Amino acids at the N- and C-terminal ends maintain the dimeric structure of the enzyme. The bottom inset shows *in vitro* polymerization assays evidencing that deletion of the first 27 N-terminal amino acid residues enhances enzymatic activity. (B) Close-up of interactions established between the N-terminus of the molecule B (blue) with the central cavity of molecule A (wheat). The first nine protein residues are disordered and not visible in the electron density. The first visible residue, P10, is located at a 18 Å of the active site. (C) Close-up of interactions established between the C-terminus of the molecule B with the fingers domain of molecule A. In panels B and C, the side chains of residues involved in intermolecular interactions are depicted as sticks and explicitly labeled. Due to the presence of the non-crystallographic symmetry (ncs) dyad interactions between the N- and C-terminus of molecule A with molecule B and those of the N- and C- terminus of molecule B with molecule A are identical.

**Fig 4:**
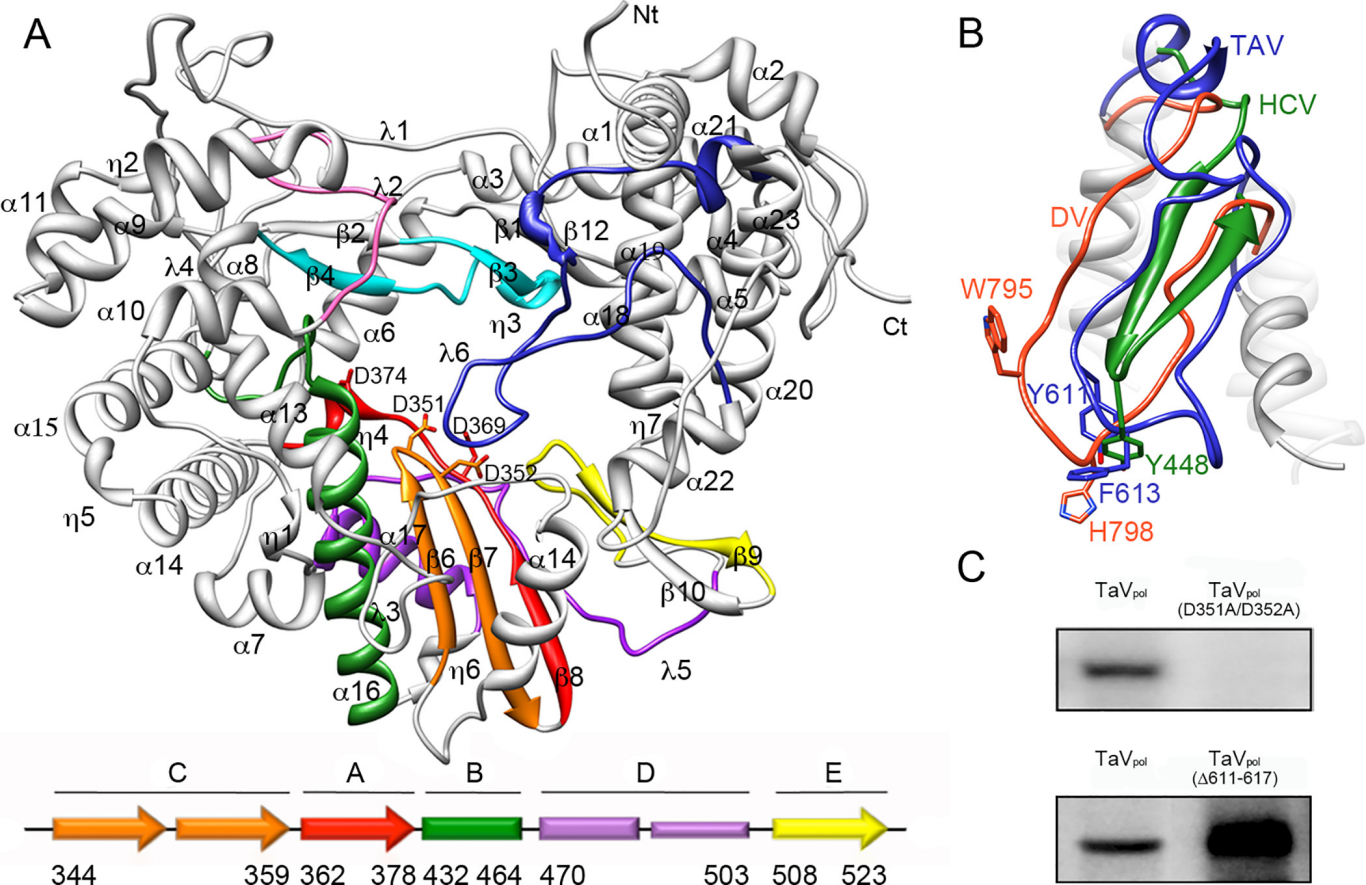
Structure of the TaV_pol_ monomer. (A) Ribbon diagram of the TaV enzyme showing the typical closed right hand architecture which encircles the seven conserved sequence motifs (A, Red; B, green; C, Orange; D, violet; E, yellow; F, cyan; G, pink). The secondary structural elements as well as the N- and C-terminal ends of the enzyme are labeled. The long λ6 loop which partially occludes the central cavity of the enzyme is highlighted in dark blue. The two aspartic acid residues of motif C (D351 and D352) and motif A (D369 and D374) are shown as sticks. The scheme (bottom panel) depicts the permuted organization of the TaV palm domain. (B) Close-up of the λ6 loop with the two aromatic side chains, Y611 and F613, shown as sticks and labeled. The structures of the equivalent loops in two distinct members of the *Flaviridae* family, HCV (green) and DV (orange) are superimposed. The HCV primer binding residue Y448, responsible for the interaction with the first nucleotide added to the newly synthesized RNA chain [19] is located close to F613 in TaV and to H798 in DV. (C) Autoradiographs of *in vitro* TaV RdRP activity, analyzed in 7% acrylamide TBE gels, showing that mutations in motif C residues D351 and D352 abolish RNA synthesis (top), and that elimination of the tip of λ6 (residues 611–617) enhances RNA polymerization (bottom).

The structure of TaV_pol_ was solved by SAD methods from Lu^3+^ derivative co-crystals to 3.0 Å resolution (Table 1) [13]. Native data was then used to complete and refine the model to a final resolution of 2.15 Å (Table 1). The crystal asymmetric unit comprises a tightly packed polymerase dimer containing 1,326 residues: from P10 to K672 of molecule A and from P10 to E674 of molecule B. Monomers A and B are almost identical, with a r.m.s deviation of 0.27 Å for the superimposition of all residues. Each monomer consists in a globular RdRP core (residues 41–648) and two terminal arms (residues 10–40 and 649–674) that extend out of the core and are involved in a number of intermolecular interactions that stabilize the dimeric structure (Fig 3). The RdRP core adopts the classical closed “right-hand” architecture consisting of fingers (helices α3-α13 and α15-α16; amino acids 41–303 and 375–443), palm (α14, β6-β8; 304–374 and α17-α18, β9-β10; 444–519), and thumb (α19-α24; 520–649) sub-domains, encircling the seven conserved motifs (A to G) that are required for substrate recognition and catalysis (Fig 4A). As expected from previous bioinformatics predictions [3,4], structural comparisons using Dali [18] show important similarities between TaV_pol_ and birnavirus polymerases. The highest hits were obtained with the IPNV (PDB id 2YIB) and IBDV (PDB id 2QJ1) RdRPs which showed Z scores of 25.4 and 21.9 and r.m.s deviations of 3.1 and 2.9 Å for the superimposition of 523 and 524 residues, respectively. Moreover, unexpected and striking resemblances were also observed when the overall TaV_pol_ architecture was compared to those of different members of the *Flaviviridae* family, with Z scores of 16.1 (Japanese encephalitis virus; PDB id 4K6M), 13.8 (DV; PDB id 4V0R) and 13.8 (HCV; PDB id 2XIZ) with r.m.s deviations of 3.3, 3.3 and 3.4 Å for the superimposition of 448, 444, and 330 residues, respectively. Similar results were obtained when the individual subdomains were superimposed (S3 Fig).

**Table 1:** Data collection and refinement statistics.

	TaV_pol_(apo)	Lu^+3^	CTP/Mg^2+^	GTP/Mg^2+^	RNA-ATP/ Mn^2+^
**Data collection**					
Space group	P2_1_2_1_2	C222_1_	P2_1_2_1_2	P2_1_2_1_2	I222
Cell dimensions					
*a*, *b*, *c* (Å)	134.97, 150.82, 100.61	154.05, 224.57, 128.29	137.70, 149.60, 100.10	137.70, 149.60, 100.11	142.51, 158.33, 217.09
Resolution (Å)*	39.6–2.15 (2.27–2.15)	63.5–3.0 (3.16–3.0)	66.9–2.25 (2.3–2.25)	66.8–2.3(2.32–2.3)	30.0–3.5(3.63–3.5)
*R*_merge_	0.12(0.29)	0.12(0.37)	0.10(0.35)	0.11(0.42)	0.15(0.31)
*I* / σI	9.0(4.1)	13.3(4.5)	9.2(3.5)	4.5(1.8)	8.3(4.0)
Completeness (%)	97.5(95.6)	98.7(96.5)	98.1(95.2)	98.6(97.6)	91.5(87.5)
Redundancy	4.6(3.0)	6.5(5.9)	5.0(3.6)	3.1(3.0)	8.1(6.9)
**Refinement**					
Resolution (Å)	39.61–2.15	63.5–3.0	66.94–2.25	30.5–2.3	30.5–3.5
No. UnicReflections	99557	44235	94439	88336	27974
† *R*_work_ / ‡ *R*_free_	17.53/21.58	18.21/24.39	19.53/22.73	17.82/22.62	25.58/29.71
No. atoms					
Protein	10526	10476	10511	10509	10514
Ligand/ion	131	54	221	174	81
Water	723	0	618	548	0
*B*-factors					
Protein	24.60	33.50	29.70	35.80	32.40
Ligand/ion	50.40	61.50	55.40	82.20	81.60
Water	29.90		33.70	36.60	
R.m.s. deviations					
Bond lengths (Å)	0.002	0.003	0.007	0.005	0.005
Bond angles (°)	0.64	0.51	1.14	0.87	1.04

† Rwork = Σhkl ||Fobs(hkl)|—|Fcalc(hkl)|| / Σhkl |Fobs(hkl)|, where Fobs and Fcalc are the structure factors, deduced from measured intensities and calculated from the model, respectively.

‡ Rfree = as for Rwork but for 5% of the total reflections chosen at random and omitted from refinement.

* Values in the parenthesis are of the highest resolution shell

**Fig 5:**
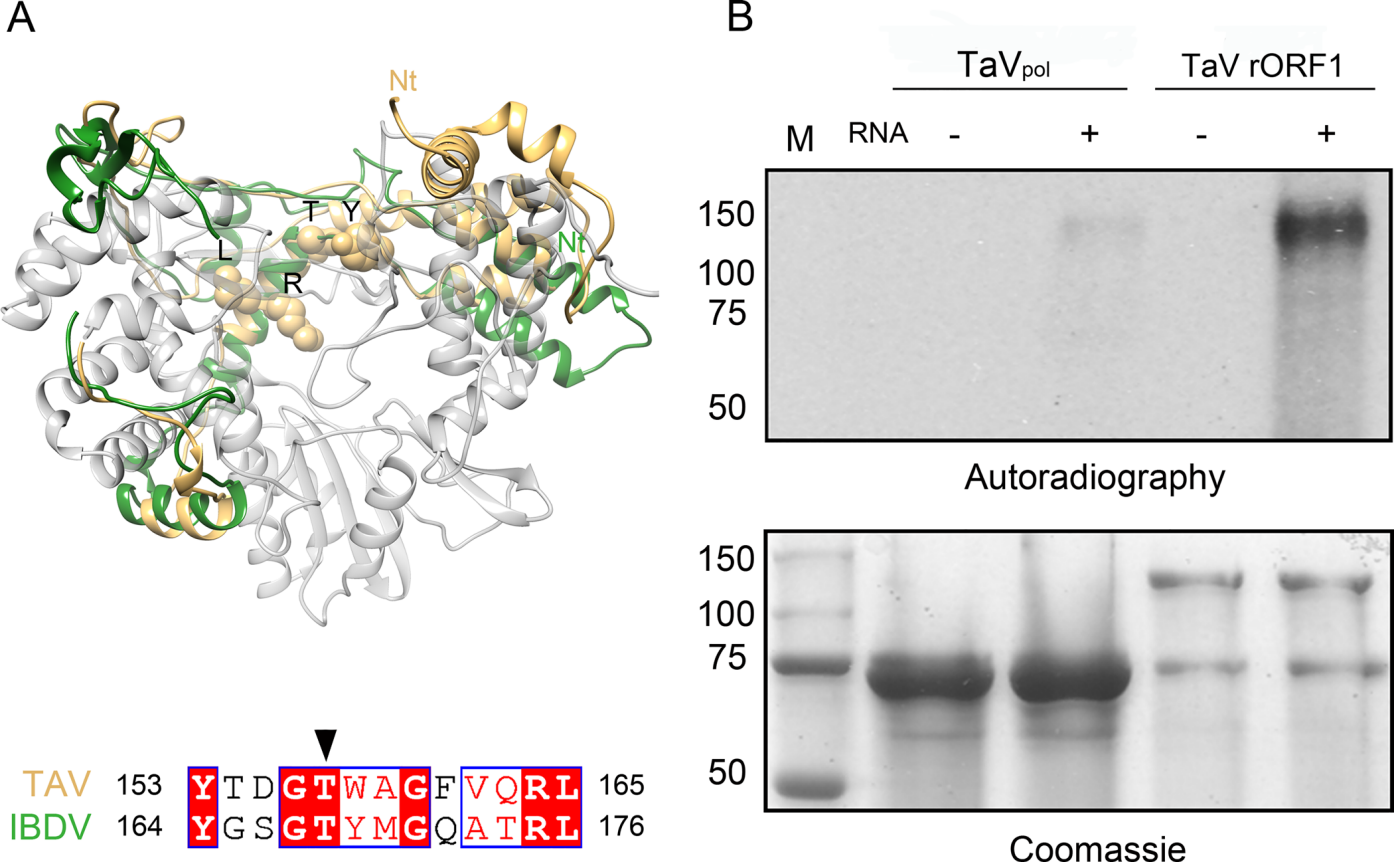
Structure of the putative VPg site. (A) Structural superimposition of the TaV polymerase N-terminus (gold) with the equivalent residues in the IBDV RdRP (green). The strictly conserved residues within the VPg signature are shown as sticks and labeled. (B) Autoradiograph of *in vitro* self-guanlylation activities of TaV_pol_ and TaV rORF1 analyzed 11% SDS-PAGE (top). Gels were also stained with Coomassie blue to assess protein loading (bottom). The position of molecular mass markers (M) is indicated (kDa) at the bottom gel.

The putative VPg signal (residues 153–165) is located at the index finger (PV nomenclature [20]), covering the α5-α6 connection and the α6 N-terminus (Fig 4A). The structure of this motif appears closely related to its birnavirus counterpart (Fig 5A) [9–11]. Upstream this motif, three helices (α3-α5) also contribute to the index finger crossing the palm sub-domain to interact with the thumb and closing the right hand structure (S4 Fig). Finally, α3 is linked by a long loop to the N-terminal helices α2 and α1 that extend outside the polymerase core. Large structural differences are observed in this N-terminal region when birnavirus and permutatetravirus are compared (Fig 5A). The self-nucleotidylation activity of the TaV enzyme was analyzed *in vitro* using both TaV_pol_ and TaV rORF1 constructs in the presence of the TaV-derived ssRNA template described above. Auto-nucleotidilation of TaV_pol_ has not been detected (Fig 5B). In addition, TaV_pol_(T157A) and TaV_pol_(S4A) mutants, where the predicted nucleotidylation residues [4, 11] were replaced by alanine, maintain levels of RNA synthesis similar to those detected with the wild type enzyme in *in vitro* polymerization assays (S5 Fig). Only the full-length TaV rORF1 retains the α-^32^P GTP radioactive signal (Fig 5B). Although more experiments are required to precisely map the guanylation site, this observation indicates that the TaV ORF1 C-terminus is essential for self-nucleotidylation.

The C-A-B permutation of the TaV_pol_ palm, with the GDD motif (residues 350–352), located at the β6-β7 hairpin, and motif A residues D369 and D374, lying at the end of strand β8, is spatially compatible with a canonical organization of the active site (Fig 4A). Similar palm architectures were found in the RdRP structures of birnaviruses [9–11].

The helical thumb of TaV_pol_ is larger than the thumb domains of other ssRNA RdRPs known to initiate replication in a primer-dependent manner as picorna- and calicivirus polymerases [2,21]. In addition, the TaV_pol_ thumb possesses a long loop (λ6; residues 591–625), protruding into the central cavity that is structurally equivalent to the priming loops of flaviviruses and bacteriophage ϕ6 [21–24] (Fig 4B). Structural comparisons show that the λ6 loop, connecting helices α20 and α22, originates from the same part of the thumb subdomain as for flaviriruses DV and West Nile Virus (WNV) but is larger and contains two secondary structural elements in its N-terminus: the short α21- and the one turn 3_10_ η8-helices (Fig 4A and 4B). The position of this element is stabilized by interactions established between different α21 residues which contact the α1 helix at the polymerase N-terminus, and between the tip of the loop (residues 613–616) with residues 301–304 and 317–320 within helix α12 and the loop α12-α13, respectively.

To further investigate the role of the λ6 loop in TaV RdRP activity, we generated a deletion mutant, TaV_pol_(Δ611–617), expected to display an open active site, lacking the putative priming platform which supports the rNTP primer during *de novo* initiation but that, in turn, may favor the accommodation of the newly synthesized dsRNA during elongation. The RNA elongation activity was then tested using the RNA template derived of the TaV 3’-UTR. Analysis of reaction products on denaturing polyacrylamide gels showed an increased activity of the TaV_pol_(Δ611–617) mutant on this template when compared to the original enzyme (Fig 4C). Comparable increased activities were also observed after similar deletions within the equivalent priming loops of HCV and DV RdRPs [25,26]. As the long ssRNA template used in these assays is able to form a fork by base complementarity that can be placed in the RdRP central cavity, the observed elongation products of this mutant would be generated by back-primed RNA synthesis. Supporting this interpretation, the *de novo* RNA synthesis on short oligonucleotide templates is abolished in the TaV_pol_(Δ611–617) mutant (S6 Fig).

### Dimeric organization of TaV_pol_

Both polymerase molecules in the asymmetric unit associate in a pseudo two-fold molecular axis. The contact surface between these two molecules, calculated using the PISA program [27], shows a total area of 6,038 Å^2^ (~11% of its total surface) and predicts a dimer stabilizing energy of ΔG_diss_ = 46.3 kcal/mol. The interface of the interaction involves: (i) the N-terminus of one molecule contacting the active site cavity of the second polymerase; and (ii) the C-terminal end of one molecule contacting the top fingers of the second one (Fig 3).

Interactions mediated by the polymerase N-terminus involve the visible part of the N-terminal end (residues 10–14), and helix α1 (residues 15–29) that extends towards the central cavity of the neighboring molecule (dyad related), contacting the finger helix α8 (residues 205–207) and the α8-α9 loop (208–225). The intermolecular interactions are mainly main-chain main-chain hydrogen bonds but also include a salt bridge between R37 and D101 (β3). The first visible residue in the electron density (P10) occupies the base of the template channel, at approximately the expected position of the first templating nucleotide, in close contact with residues 209–211 (Fig 3B). These contacts involve a total of 38 residues, covering a surface of 2,725 Å^2^ with an energy ΔG_diss_ = 16.5 kcal/mol. Equivalent crystals have been obtained after the enzymatic cleavage of the N-terminal hexa-histidine tag. Unfortunately, the resulting structure did not revealed additional information about the positioning of the first nine protein residues.

In order to explore the functional role of the polymerase N-terminus in close contact with the template channel of the neighboring enzyme, we designed a TaV rORF1 mutant, TaV rORF1(Δ27), lacking the first 27 N-terminal residues (Fig 1). Surprisingly, TaV rORF1(Δ27) does not undergo the cleavage into the 75 kDa polypeptide observed in the full-length protein and, in addition, it is organized as a monomer in solution (Fig 1C). Polymerization assays performed with this mutant as well as with the TaV_pol_(Δ27-Δ657) construct show that the elimination of the first 27 residues that prevents dimer formation also causes a significant increase on RNA synthesis (Figs 3A, bottom inset, and S7).

The TaV_pol_ C-terminus is formed by a long arm (residues 649–674) that extends along the finger helices α8, α9 and α14 at the external surface of the protein (Fig 3C). The C-terminal-mediated interactions include 43 residues, forming a contact surface of 3,313 Å2, with a ΔG_diss_ = 17.2 kcal/mol.

TaV_pol_ dimers were also observed by negative staining transmission electron microscopy (S8 Fig), indicating that the dimer structure, first observed in crystals, is stable in solution and maintained even at very low protein concentrations.

### Structure of TaV_pol_ in complex with a ssRNA template and incoming rNTPs

TaV_pol_-ssRNA-rNTP complex co-crystals were obtained after incubation of TaV_pol_ with the oligonucleotide template 5’-CCCAUUCGACUCCUG, ATP, CTP and MnCl_2_. This complex crystallized in the space group I222 with one TaV_pol_ dimer in the asymmetric unit. The structure was solved by Molecular Replacement and refined to 3.5 Å resolution (Table 1). Structural comparisons between unbound and ssRNA-rNTP-bound enzymes revealed two significant conformational changes: (i) a ∼7˚ rotation of one monomer with respect to the other in the dimer; and (ii) a conformational rearrangement of the polymerase N-terminus, resulting in a subtle opening of the central cavity that facilitates template entry (S9 Fig).

**Fig 6:**
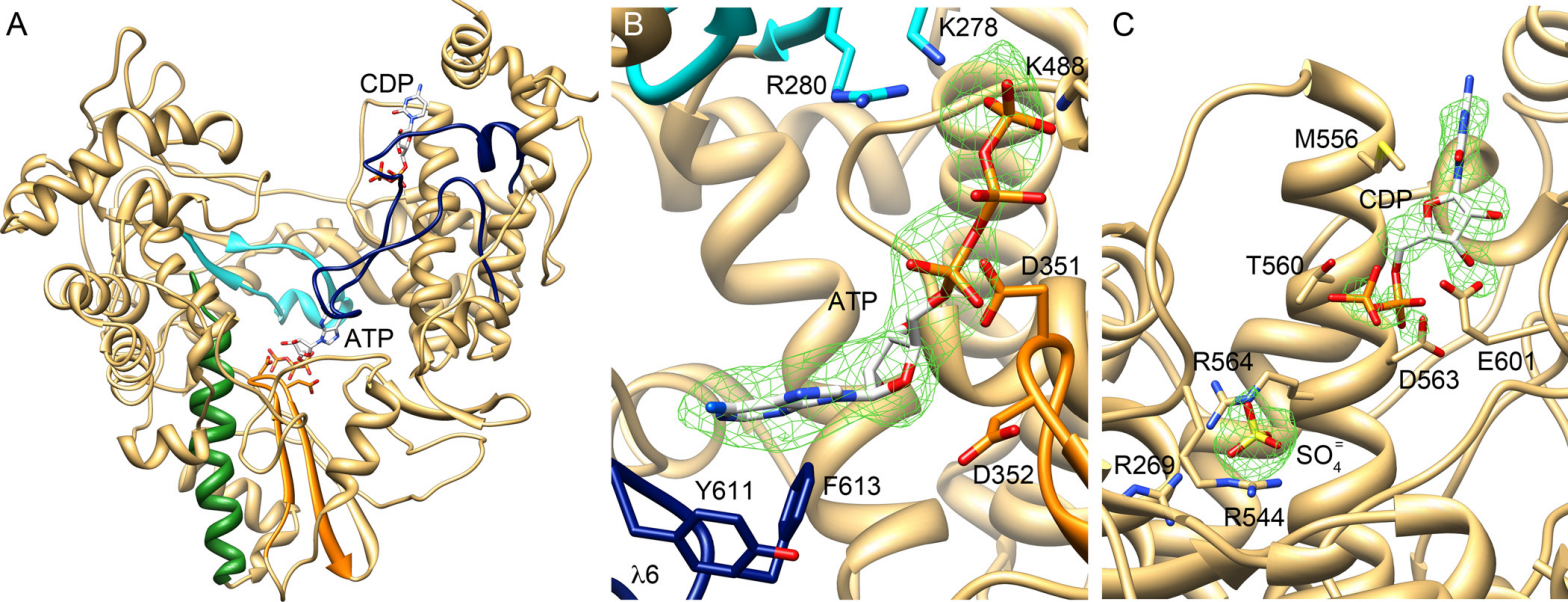
Structure of TaV_pol_-ssRNA-rNTP and TaV_pol_-rNTPcomplexes. (A) Location of the two binding sites for incoming rNTP molecules found in the different crystal structures. Motif C is highlighted in orange with the catalytic residues D351 and D352 shown as sticks. (B) Close-up of the ATP molecule bound to the TaV_pol_ active site with the adenine base firmly stacked on the Y611 and F613 residues in the priming loop λ6 (blue). The ribose moiety interacts with the catalytic D351 residue of motif C (orange), and the triphosphate is involved in salt bridges with K278 and R280 in the fingers motif F (cyan) and K448 of motif D. The 2Fo-Fc electron density map around the ATP molecule is displayed at a contour of 1σ (green mesh). (C) Unexpected binding site for a CTP nucleotide in the thumb domain of TaV_pol_. The 2Fo-Fc electron density map (1σ) is shown as a green mesh. An additional strong peak of electron density, found in the vicinity of the NTP binding site, was interpreted by the presence of one sulfate ion from the crystallization solution. This sulfate appears tightly bound to the side chains of three R residues.

The structural analysis of this complex revealed the presence of an extra density at the polymerase active site in one of the two molecules of the crystal asymmetric unit (Molecule B). This density, was interpreted as the presence of a bound ATP molecule, with the ATP base tightly stacked to residues Y611 and F613 of loop λ6 and the triphosphate moiety occupying the nucleotide entry tunnel, contacting the basic residues R280, K278 of motif F and K488 of motif D (Fig 6A and 6B).

Unfortunately, the electron density corresponding to the ssRNA template was too weak and discontinuous to allow the building of an accurate model. However, strong peaks were detected along the template channel of the polymerase that would correspond to the phosphate moieties of the oligonucleotide bound in a similar orientation to that of templates derived from the superimposition of available RdRP-ssRNA complexes onto the TaV enzyme (S10 Fig).

Furthermore, an additional peak of electron density was also seen in close contact with the motif B residues T443 and T444, far from the trajectory of the putative template phosphodiester chain (S10 Fig). The X-ray analysis of a second TaV_pol_-ssRNA complex indicated that this density would correspond to the template base at position 3’ that overshoots its predicted binding site in front of the incoming rNTP, appearing tightly packed to these motif B residues. Unfortunately, only a partial data set could be collected from these crystals (53.8% completeness to 3.1 Å resolution; S10 Fig). Motif B contains a number of S/T residues strictly conserved in RdRPs that are involved in template binding and translocation of the newly synthesized dsRNA [28–29]. The TaV_pol_-RNA complex suggests that these motif B residues might also serve as a binding site for the terminal base of the template in a pre-initiation stage. To assess the role of these conserved residues on RNA synthesis, T443 and T444 were substituted by Ala. The RdRP activity of the mutant enzyme was analyzed *in vitro*, showing that the T→A replacements at positions 443 and 444 of TaV_pol_ completely abolish RNA synthesis (S10 Fig).

### An unusual nucleotide binding site in the thumb domain of the TaV RdRP

TaV_pol_-GTP and TaV_pol_-CTP co-crystals were also obtained in presence of MgCl_2_ and the corresponding structures solved to 2.3 Å and 2.25 Å resolution, respectively (Table 1). In both cases, clear electron densities were observed for the triphosphate moieties interacting with electropositive residues at the rNTP tunnel. However, the corresponding nucleoside parts were disordered. In addition, these structures revealed a second nucleotide binding site in a totally unexpected region, a cavity inside the thumb subdomain, at about 30 Å from the active site (Fig 6C). In this position, the nucleoside moieties of the bound NTPs contact residues M556, T560 and D563 from the α19 helix and to E601 from the λ6 N-terminus, whereas the triphosphate moieties remain partially exposed to the solvent, appearing mostly disordered. It should be noted that the nucleotides are bound next to a strong electropositive region, residues R564, R545 and R269, also containing an extra density peak, interpreted as a sulfate molecule derived from the crystallization solution (Fig 6C). Noteworthy, this sulfate is present in all other analyzed TaV_pol_ structures.

## Discussion

As proposed in previous theoretical studies [3], the crystal structures of the non-canonical TaV RdRP show the conservation of the architecture of the active site. The biochemical characterization of the RdRP activity of TaV_pol_ also confirms that the observed relocation of the catalytic motif C does not affect enzyme activity (Fig 4C). This observation provides the first experimental evidence of polymerization activity in a non-canonical RdRP from a +ssRNA virus. *In vitro* polymerization assays also demonstrate that TaV_pol_ is able to incorporate nucleotides on ssRNA templates of different size and sequence, and in the absence of primer (Fig 2). Similar activities have been described in polymerases from the *Flaviviridae* family [16]. The kinetics profile obtained, exhibiting a very low activity at the beginning of the reaction, is also similar those observed with flaviviral RdRPs, e.g. DV and HCV, where elongation products are only detectable from incubation times of ca. 30 min onwards (S2 Fig). These enzymes appear to require the preceding period to form the *de novo* initiation complexes [16, 26,30–31].

The TaV_pol_ thumb harbors loop λ6 closely resembling flavivirus and bacteriophage ϕ6 priming loops, known to serve as a support platforms for the *de novo* initiation of RNA synthesis. For this purpose, the presence of an aromatic side chain at the tip of the loop seems to be essential to orientate the base of the priming nucleotide (i.e. Y660 in ϕ6, Y448 in HCV, W795 and H798 of DV, and W800 and H803 in WNV [19, 21–23]). Loop λ6 possesses residues Y611 and F613 at equivalent positions (Fig 4B). Indeed, the structure of the HCV NS5B ternary complex, mimicking a primed initiation complex, shows the aromatic ring of Y448 stacked against the priming nucleotide [19]. Also the TaV_pol_-ssRNA-ATP complex, determined in this work, shows the ATP substrate resting on loop λ6, with the adenine base contacting both Y611 and F613 side chains (Fig 6B). Altogether, our the structural and biochemical data indicate that the TaV_pol_ is able to perform RNA synthesis using a *de novo* initiation mechanism akin to those found in flaviviruses and in bacteriophage ϕ6 [16,24], with the λ6 loop acting as an initiation replication platform in an analogous way to the β-flap in HCV [19,25], the priming loops of DV and WNV [23,32], and the β-thumb region of the bovine viral diarrhea virus [33].

Besides the λ6 loop, the polymerase N-terminus would also play a regulatory replication-initiation role, controlling dimerization. The presence of TaV_pol_ homodimers, both in crystals and in solution, correlates with previous observations gathered with other polymerases. RdRP oligomerization has been described in different virus families, including calici- [34], picorna- [35–38], flavi- [39–41] and birnavirus [11]. Therefore, although its structural basis remains still unclear, oligomer formation appears to be a common feature among RdRPs which directly affects enzyme activity. Surprisingly, the pre-active form of the eukaryotic RNA Pol I is also dimeric and shows a regulatory peptide bound to the template-binding site [42,43]. Structures presented in this report show that TaV_pol_ dimers are stabilized by mutual interactions established between the N-terminus of one molecule and the active site cavity of its interacting neighbor (Fig 3B). Despite the C-termini of both molecules also appear to contribute to stabilize the dimer, studies performed with deletion mutants TaV rORF1(Δ27) and TaV_pol_(Δ27-Δ657) show that only the first interaction is critical for maintaining the dimeric structure (Fig 1C). In addition, biochemical data show that the removal of the first 27 N-terminal residues from either TaV_pol_ or the full-length rORF1 protein significantly boosts polymerase activity (Figs 2 and S7). Taken together, these data suggest that dimer formation provides the means to regulate the polymerization activity. The presence of the neighboring polymerase N-terminus would compete with the correct positioning of the RNA template, thus affecting enzyme performance. In fact, the structure of the TaV_pol_-ssRNA-ATP complex shows a conformational change, consisting in displacement of the N-terminal arm towards the outside of the molecule (S9B Fig). This movement, occurring in concert with a rotation of the two molecules in the TaV_pol_ dimer, results in the opening of the cavity for template entry.

**Fig 7:**
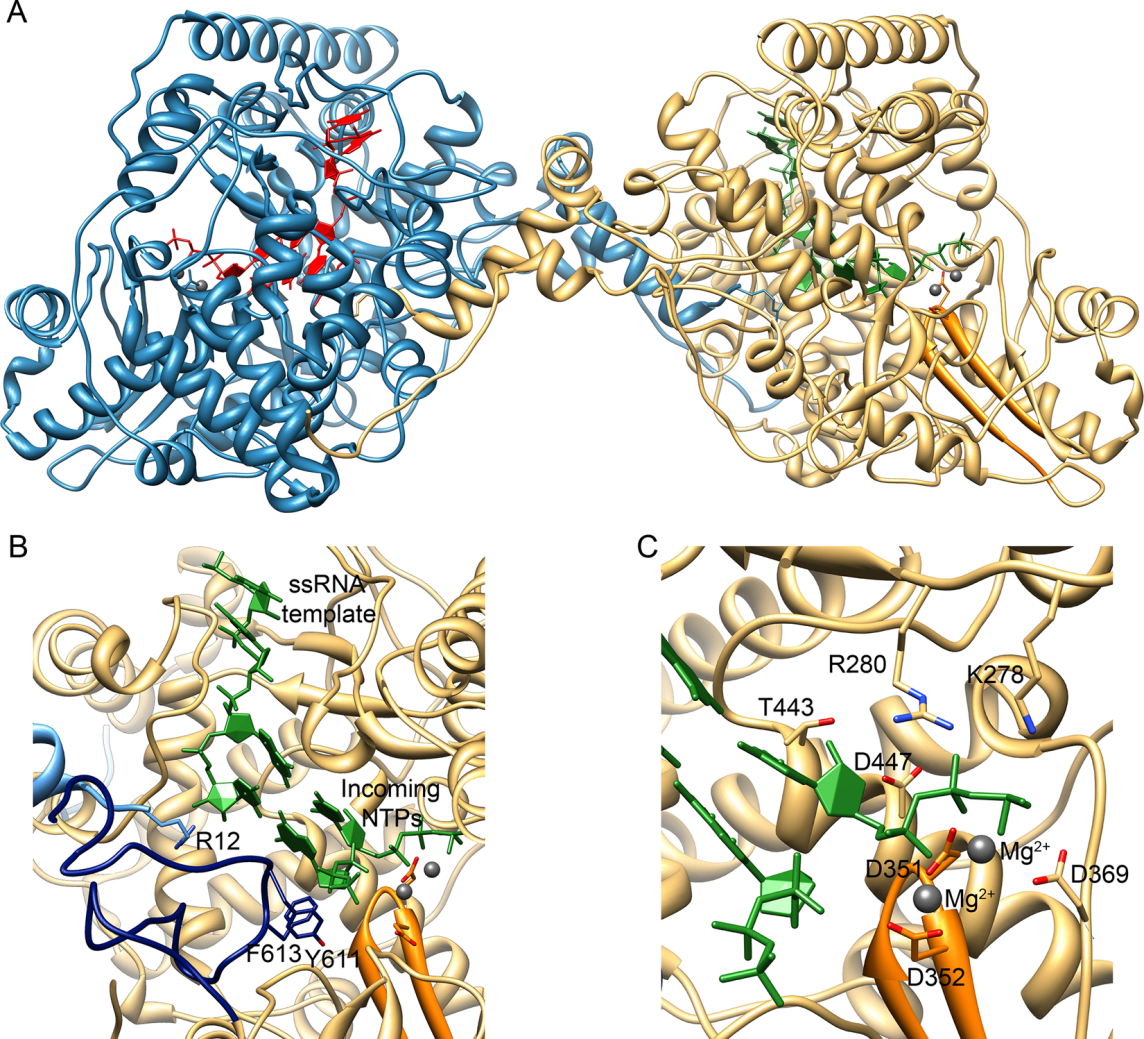
Model for the *de novo* initiation of RNA synthesis by the TaV_pol_ dimers based on the ϕ6 replication-initiation complex (PDB ID 1HI0). (A) Overall view of the TaV dimers with bound templates and NTPs shown in red and green for the molecules A and B, respectively. (B) Close-up of the polymerase active site with the catalytic motif C shown in orange, the λ6 loop in dark blue and the N-terminus of the neighboring polymerase molecule in cyan. The side chains that appear to be involved in critical functions are shown as sticks and explicitly labeled. (C) Close-up of the active site showing the position of incoming rNTP and metal ions (grey balls). Polymerase side chains in direct contact with the modeled nucleotide and ions are shown as sticks and explicitly labeled.

The phosphate molecules, visible in the TaV_pol_-ssRNA-ATP complex co-crystals, associate with the fingers domain in the template channel, likely representing the template strand being directed to the catalytic site. The basic R12 side chain of the rearranged N-terminus appears in contact with the phosphate that would correspond to the t+1 nucleotide, close to the active site (S10 Fig), suggesting a regulatory role of this region during the first steps of the *de novo* replication initiation process. R12 is also in close contact with the templating (t+1) nucleotide in the putative initiation model (Fig 7) generated by the superimposition of the template and incoming rNTPs of the bacteriophage ϕ6 polymerase [24] onto the TaV_pol_ active site. In addition, this modeling suggests that *de novo* polymerization initiation is compatible with TaV_pol_ dimerization (Fig 7). Moreover, it is known that *de novo* initiation platforms block the path of the newly synthesized dsRNA once it reaches two or three nucleotides in length. At this point, the protein must undergo a conformational change to assist the exit of recently synthesized dsRNA during chain elongation [19, 24–25]. The TaV_pol_ structure indicates that λ6 is the only element that should be re-organized in order to facilitate the opening of the central cavity to accommodate the elongation product. The proximity of the N-terminal arm of the neighboring polymerase to λ6, and the observed interaction between R12 and the templating nucleotide in the modeled initiation complex (Fig 7), suggest a key switching role of the N-terminus in the initiation-to-elongation transition.

### Evolutionary implications

The structure of TaV_pol_ confirms the existence of a close relationship between birnaviruses (dsRNA) and permutotetraviruses (ssRNA) RdRPs, as previously predicted by sequence analyses [3]. In addition, it reveals the presence of unexpected elements (i.e. the λ6 loop and a terminal arm) controlling RNA synthesis activity. These elements are present not only in this particular enzyme but also in other RdRPs performing priming independent replication initiation such as those of flaviviruses with which it also shares functional characteristics.

Although at this point is not feasible to establish whether our findings reflect convergent or divergent evolution, the striking structural and functional similarities shared by permutotetra- and flavivirus RdRPs, reported here, constitute the first evidence about the existence of an evolutionary relationship connecting the polymerases of these two apparently distant virus groups.

Three alternative scenarios for the evolution of polymerases harboring permuted palms can be envisaged: (i) a circular permutation giving rise to a non-cannonical RdRP might have taken place in a population of +ssRNA viruses likely belonging to the flavi-like Group II. Thereafter, both canonical and non-canonical polymerases might have coexisted until the advent of the dsRNA birnavirus ancestor; (ii) the horizontal transfer of an ancestral permuted RdRP gene between members of two otherwise unrelated +ss and dsRNA virus lineages [44]; and (iii) the occurrence of two independent circular permutation events in dsRNA and +ssRNA virus lineages. Although at first these three genetic scenarios seem equally feasible, the remarkable resemblances between capsid proteins of birnaviruses and members of the old *Tetraviridae* family [45–47] advocate the first alternative. Indeed, this hypothesis might entail the existence of a common birna-, tetra- and flavivirus ancestor RdRP polypeptide.

## Materials and Methods

### Protein expression and purification

Expression and purification of TaV rORF1 and TaV_pol_ were previously reported [13]. Briefly, a recombinant baculovirus (rBV), expressing the whole ORF1 polypeptide of *Thosea asigna* virus (1257 residues, 140 kDa; GenBank accession number AF282930.1) fused to a 6xhistidine tag and containing the TEV protease recognition site (hTaV ORF1), was generated, according to the Bac-to-Bac protocol (Invitrogen). H5 cells (Invitrogen) were infected with the hTaV ORF1 rBV, harvested at 72 h post-infection, washed twice with PBS, resuspended in lysis buffer (50 mM Bis Tris pH 6.8, 500 mM NaCl, 0.1% Igepal CA-630) supplemented with protease inhibitors (Complete Mini; Roche), and maintained on ice for 20 min. After 20 min centrifugation (13,000xg) at 4°C, supernatants were collected, and subjected to metal affinity chromatography batch purification using a Co^2+^ affinity resin (TALON, Clontech). Resin-bound hTaV ORF1 was eluted with elution buffer (50 mM Bis Tris pH 6.8, 500 mM NaCl, 500 mM imidazol). SDS-PAGE showed that the purified polypeptide was partially cleaved, resulting in a product of ~75 kDa (Fig 1). The recovered product was analyzed by mass spectrometry (MALDI-TOT/TOF) to assess for the integrity of the RdRP domain, included within the first 674 amino acids of the TaV ORF1. The resulting polypeptide was further purified by size exclusion chromatography on a Superdex 200 HR 10/300 column (buffer 50 mM MES pH 6.0, 500 mM NaCl, 10% glycerol and 5 mM DTT). Finally, the purified TaV_pol_ was pooled and concentrated to 10 mg/ml.

In addition to the rORF1 and TaV_pol_, other protein versions harboring either point mutations TaV_pol_(S4A), TaV_pol_(T157A), TaV_pol_(D351A/352A) or TaV_pol_(T443A/444A) or deletions TaV rORF1(Δ27) TaV_pol_(Δ27-Δ657) or TaV_pol_(Δ611–617), were also generated (Fig 1), expressed and purified in a similar fashion as for the wild-type protein, lacking only the size exclusion chromatography step. In order to obtain the intact TaV rORF1 protein, 150 μM leupeptine hemisulfate (Apollo Scientific) was added both in the Hi5 cell culture and in the lysis buffer during the purification process that was performed in the conditions described above.

For biochemical analysis, and once in the crystallization of the apo-form of the enzyme, proteins were treated with TEV protease to eliminate the recombinant tag.

### Preparation of the ssRNA templates

ssRNA oligonucleotides of 6-, 12- and 16-nts corresponding to the 3’-end of the TaV 3’-UTR and to heterologous sequences of 13- and 25-nts length were purchased (Biomers.net). The 311-nts ssRNA template, formed by the fusion of a heterologous sequence (171-nts) to the 5’-end of a 140-nts fragment corresponding to the 3’-end region of the TaV 3’-UTR [4], was produced by *in vitro* transcription, using as template the pRSET-A/TaV-3’UTR plasmid described below. A fragment corresponding to the last 140-nts of the TaV 3’-UTR flanked by BamHI and EcoRI restriction sites was synthesized *in vitro* (GeneScript). After restriction with BamHI and EcoRI, the fragment was cloned into the pRSET-A vector previously digested with the same enzymes. The resulting plasmid, pRSET-A/TaV-3’UTR, was linearized by digestion with EcoRI and used as a template for *in vitro* transcription reactions using a commercial kit (RiboMax, Promega) according to the manufacturer specifications. ssRNA was isolated from agarose gels, recovered by electroelution (International Biotechnologies Inc.), precipitated with ammonium acetate/ethanol, and resuspended in DEPC-treated H_2_O.

The unrelated template sequence of 320-nts ssRNA was obtained as described above by fusing the same 171-nts heterologous sequence to the 5’-end of the 149-nts fragment, corresponding to the 3’-end region of RNA1 of the SJNV genome [48].

### Polymerase activity assays

Polymerase activity assays were performed following a previously described protocol [49] with minor modifications. Briefly, reaction mixtures containing 1 μg of purified TaV_pol_ and TaV rORF1 wild type or variants (Fig 1), were prepared in 40 μl of transcription buffer (50 mM MES pH 6.0, 100 mM NaCl, 5 mM MgCl_2_, 10% glycerol, 1 mM DTT, 1 mM rATP, rGTP and rCTP, 0.02 mM rUTP, 20 units of RNasin, and 10 μCi [α-^32^P] rUTP), supplemented with 5 μl of ssRNA+ template (0.2 mg/ml). Samples were incubated at 35°C for 1 h, or the time indicated in each experiment, heated to 100°C for 3 min to stop the reaction, and subsequently digested with 0.2 mg/ml of Proteinase K for 1 h at 37°C. Reaction products were mixed with loading buffer (10 mM Tris-HCl pH 7.5, 15% Ficoll 400, 50 mM EDTA, 0.03% orange G, 0.03% bromophenol blue, and 0.03% xylene cyanol) supplemented with 80% formamide, incubated at 60°C during 10 min and subjected to electrophoresis in 7% polyacrylamide TBE (90 mM Tris, 64.6 mM boric acid, and 2.5 mM EDTA, pH 8.3) gels. Radioactive signals were detected with a Storm gel imaging system (Molecular Dynamics). Results were analyzed and quantified with Image Quant software (Molecular Dynamics). Additionally, filter binding and liquid scintillation counting were used to monitor RdRP activity. Aliquots of the reactions (20 μl) were spotted onto DE-81 filter discs (Whatman). Filters were dried, washed three times with 50 mM K_2_HPO_4_ pH 7.4 and once with ethanol. After drying, filters were immersed in liquid scintillation fluid, and [α-^32^P]UTP incorporation measured in counts per minute using a liquid scintillation counter (Wallac).

### Nucleotidylation assays

Reaction mixtures containing the TaV_pol_ or the full-length TaV rORF1 supplemented or not with 5 μl of a ssRNA template (0.2 mg/ml) were performed for 10 min under optimal conditions (50 mM MES pH 6.0, 100 mM NaCl, 5 mM MgCl_2_, 10% glycerol, 1 mM DTT, 1 mM ATP, UTP and CTP, 0.02 mM GTP, 20 units of RNasin, and 10 μCi [α-^32^P] GTP at 35°C). Reaction products were subjected to 11% SDS-PAGE, and recorded by autoradiography following an 8 h exposure period.

### Crystallization of the TaV_pol_, apoprotein and substrate-bound complexes

Crystals of TaV_pol_, apo-form and the Lutetium derivative were obtained by the sitting-drop vapor diffusion method as previously described [13]. The RNA oligonucleotide (sequences 5’-CCCAUUCGACUCCUG) was used as a template to form the binary complex in a 1:1.5 TaV_pol_:RNA molar ratio with 1 mg/ml TaV_pol_ and incubated at 30°C for 30 min in a buffer containing 50 mM MES pH 6.0, 200 mM NaCl, 10% glycerol and 5 mM DTT. Samples were then concentrated using Centricon 30K tubes (Millipore) to a final protein concentration of 10 mg/ml. In order to obtain the ternary complex, the TaV_pol_-CCCAUUCGACUCCUG complex was also incubated with mixtures of rCTP and rATP, in presence of MnCl_2_, to a final concentration of 5 mM. Crystals of the binary and ternary complexes were obtained using the sitting-drop vapor diffusion technique at 20°C, by mixing 0.5 μl of complex with 0.5 μl of crystallization buffer (12% PEG 8K and 750 mM Li_2_SO_4_). All crystals were transferred to a cryoprotectant solution containing 20% glycerol in the crystallization buffer, and then were flash frozen in liquid nitrogen.

### Data collection and structure solution

All diffraction data sets were collected at 100 K from single crystals using synchrotron radiation. Native TaV RdRP data were collected up to 2.1 Å resolution on ADSC Q4R detector at the ESRF beam line ID14EH4 (λ = 0.98). Lutetium derivative data (3.0 Å resolution) were collected on ID23.1 beam line at a wavelength corresponding to the lutetium absorption edge (λ = 1.3404) [13]. Data from the CTP, GTP and CCCAUUCGACUCCUG/CTP/ATP/Mn^2+^ complexes were also collected at the ESRF ID14EH4 beamline (λ = 0.98) to resolutions of 2.25 Å, 2.3 Å and 3.5 Å, respectively (Table 1).

Diffraction images were indexed and integrated using iMOSFLM [50–52] and XDS programs and scaled, merged and reduced with SCALA from the CCP4 program suite [52]. The structure of the isolated enzyme was determined by a single-wavelength anomalous diffraction (SAD) phasing, combined with Molecular Replacement of the partial models obtained as implemented in the Auto-Rickshaw pipeline [53]. A partial model (containing 629 residues for each of the two molecules in the asymmetric unit) automatically produced by the program BUCCANEER [54] was then used for phase improvement and model completion using the MRSAD protocol available in Auto-Rickshaw [55]. Manual model rebuilding and sequence assignment, performed with program COOT [56], was alternated with cycles of automatic refinement by using programs REFMAC5 [57] and PHENIX [58]. Native data was then used to complete and refine the final model of the RdRP apo-form (Table 1).

The structures of the TaV_pol_ complexes were obtained by Molecular Replacement, using the coordinates of the unliganded polymerase as search model, using the program Phaser (CCP4i). Refinement and manual model rebuilding of the different complexes proceeded as for the unliganded crystals. Data refinement statistics are listed in Table 1.

Calculation of contact surfaces and volumes was performed with programs VADAR [59] and MOLE2.0 [60]. Illustrations were prepared with Chimera [61].

### Data deposition

The atomic coordinates and structure factors have been deposited in the Protein Data Bank, www.pdb.org (PDB ID codes 4XHA, 4XHI, 5CX6, 5CYR).

## Supporting Information

S1 Fig Effect of different reaction conditions on polymerase activity. Autoradiograms of 7% TBE-PAGE corresponding to polymerization reaction products of TaV_pol_ where the (A) pH of the reaction buffer (50 mM citrate pH 4.5; 50 mM MES pH 6; 50 mM NaH2PO4/Na2HPO4 pH 7; 50 mM Tris-HCl pH 8; and 50 mM Bicine pH 9), (B) temperature, (C) ionic strength, (D) protein concentration or (E) GTP concentrations were changed. Reactions were performed for 120 min using standard conditions except the tested variable.
(TIF)

S2 Fig Polymerization kinetics of TaV_pol_. (A) Autoradiography of a 7% TBE PAGE corresponding to polymerization products of TaV_pol_ generated under optimal reaction conditions. The reaction was stopped at different incubation times. (B) The graph corresponds to normalized liquid scintillation determination of polymerization reactions products. Each point corresponds to the average value of quantifications from three independent experiments.
(TIF)

S3 Fig Comparisons of TaV_pol_ with birna- and flaviviruses polymerases. (A) Cα tracing of the TaV enzyme (gold) with the IBDV (left panel; green) and DV (right panel; dark blue) RdRP structures superimposed. (B) Ribbon diagrams of the individual, fingers (top), palm (middle) and thumb (bottom) sub-domain superimpositions.
(TIF)

S4 Fig Finger-thumb interactions stabilizing the closed right hand conformation of TaV_pol_. Top view of the structures closing the RdRP domain (left panel) and zoom of the back view. The interacting secondary structural elements are depicted in different colors. Extensive interactions are found between the finger helices α4 and α5 and the thumb region formed by α19, the α19-α20 loop and α21. Furthermore, the N-terminal helix α2 and the α2-α3 loop contact the thumb helices α19 and α22. This large interdomain interface results in a robust closing of the central cavity of the enzyme.
(TIF)

S5 Fig Effect of the mutations T157→A and S4→A on TaV RdRP activity. Autoradiograms of *in vitro* RdRP activity of TaV_pol_(S4A) and TaV_pol_(T157A) analyzed in 7% acrylamide TBE gels, showing that the polymerase mutants harboring substitutions in the predicted nucleotidylation residues, maintain the levels of RNA synthesis similar to those found in the non-mutated enzyme.
(TIF)

S6 Fig TaV_pol_(Δ611–617) mutant is inefficient to initiate RNA synthesis on short templates. Autoradiography of the polymerization products analyzed in a 7% acrylamide TBE gel. The first and second lines correspond to the activity of TaV_pol_ using of 16- (5’-AACCUUUUUCCACGCG) and 12-nucleotide (5’-UUUUUCCACGCG) long ssRNA templates, respectively. The third and fourth lanes correspond to the activity of TaV_pol_(Δ611–617) on the same 16- and 12-nucleotide templates. Reactions were performed in presence of 50 mM MES pH 6.0, 100 mM NaCl, 5 mM MgCl_2_, 10% glycerol, 1 mM DTT, 1 mM ATP, UTP and CTP, 0.02 mM GTP, 20 units of RNasin, and 10 μCi [α-^32^P] GTP, and incubated for 45 min at 35°C.
(TIF)

S7 Fig Comparative kinetics of the TaV rORF1 and TaV rORF1(Δ27) polymerization activities. Autoradiograms of *in vitro* RdRP activities of full-length TaV rORF1 (top) and TaV rORF1(Δ27) (bottom). The reaction was stopped at different incubation times and the reaction products were analyzed in 7%TBE-PAGE and recorded after an exposure time of 8 h. Reactions were performed in presence of the ssRNA template of 311-nts harboring the TaV 3UTR’ sequence. The bottom image shows an 11% SDS-PAGE stained with Coomassie blue of the two proteins, used as protein loading controls.
(TIF)

S8 Fig Electron micrograph of the purified TaV_pol_. A sample of the purified enzyme was negatively stained with 2% (w/v) uranyl acetate. Red and blue circles indicate dimer and monomer respectively. Scale bar, 3 nm.
(TIF)

S9 Fig Superimpositions of the apo-form, RNA-ATP-bound and Lutetium-bound structures of TaV_pol_. (A) The apo-form of TaV_pol_ is shown in cyan, the RNA-ATP-bound enzyme in magenta and the Lutetium derivative in gold. One monomer of the RNA-ATP-bound structure shows a -7° rotation around the Z axes with respect to the same position of the apo-form TaV_pol_. The same monomer of the Lutetium structure shows a 22° and -18° rotation around Z and X axes, respectively, in comparison to the apo-form enzyme. The structural comparisons of individual monomers also show a subtle opening of the central channel. (B) Different conformations adopted by the TaV_pol_ N-terminal ends in the apo structure (blue and cyan) and in the RNA-ATP complex (red and pink). In the TaV_pol_-RNA-ATP complex, the polymerase N-terminus points outwards from the central cavity leaving enough space to accommodate a ssRNA template, modeled in stick representation.
(TIF)

S10 Fig Electron densities in the template channel of the TaV_pol_-ssRNA complexes. (A) View of a σA-weighted |Fo|-|Fc| electron density map (3.5σ) around the TaV_pol_ template channel in the TaV_pol_-CCCAUUCGACUCCUG-ATP complex. The strong peaks of electron density (purple mesh) can be easily interpreted as the phosphate groups of a short oligonucleotide bound to the channel, in good agreement with template models derived from the superimposition of available RdRP-RNA complexes onto the TaV enzyme. The modeled template (shown as sticks in atom type color) was obtained by the superimposition of the HCV NS5B replication initiation complex (PDB ID 1WTA) onto the TaV_pol_ active site. (B) A partial data set from other complex co-crystals, the TaV_pol_-GUAUACUACACCCAUUCGACUCCUG complex, have been obtained and analyzed (space group I222; a = 143.4, b = 159.0, c = 218.1 Å, with one TaV_pol_ dimer in the crystal asymmetric unit). The X-ray data was collected using synchrotron radiation at the Swiss Light Source, PXI beam line. Unfortunately, these crystals were extremely sensitive to radiation and died before completing data collection (53.8% completeness at 3.1 Å, R_merge_ = 6.8%). Attempts to merge data for different crystals failed due to the lack of isomorphism. The structure was solved by molecular replacement, using the coordinates of the unliganded TaV_pol_ as search model. Analysis of the electron density maps revealed the presence of a partially ordered extra electron density to position a stretch of four nucleotides, most probably the 3’-end (5’-CCUG) of the template, occupying the template binding channel of the two polymerases molecules of the crystal asymmetric unit. Model refinement was performed with the program REFMAC5 [57] applying non-crystallographic symmetry restraints to the two protein molecules in the asymmetric unit. Automatic refinement was alternated with manual model rebuilding using coot [56]. The final refinement cycles converged to an R_work_ of 22.3%, R_free_ = 25% with good stereochemistry (r.m.s. deviations of bond lengths 0.004Å, bond angles 0.9°). The image shows a view of the template channel with the refined coordinates of the bound tetranucleotide modeled inside. Polymerase residues directly contacting the RNA are explicitly labeled. The template base at position 3’ appears tightly packed to motif B residues T443 and T444. A σA-weighted 2ІFoІ-ІFcІ map, contoured at 1.2σ, of the tetranucleotide template is shown as a green mesh. Inset corresponds to an autoradiograph of *in vitro* polymerase activity of TaV_pol_, analyzed in 7% acrylamide TBE gels, showing that mutations in residues T443 and T444 abolish RNA synthesis.
(TIF)
