# Supplementary figures and images for: The Structure of the RNA-Dependent RNA Polymerase of a Permutotetravirus Suggests a Link between Primer-Dependent and Primer-Independent Polymerases
Source: PLoS Pathog. 2015 Dec 1;11(12):e1005265. doi: 10.1371/journal.ppat.1005265 (PMC4666646; doi:10.1371/journal.ppat.1005265)

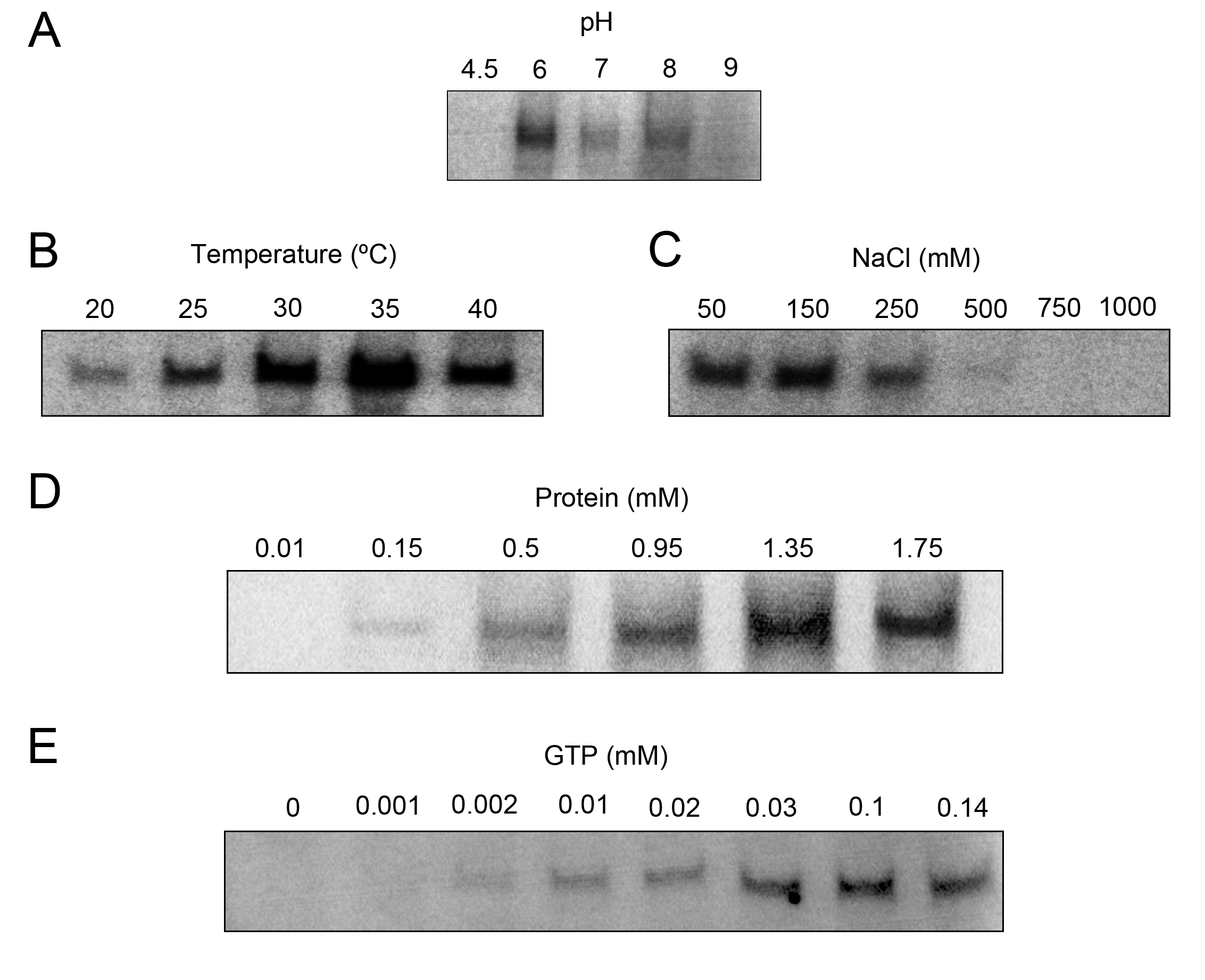

Supplement: S1 Fig — Autoradiograms of 7% TBE-PAGE corresponding to polymerization reaction products of TaVpol where the (A) pH of the reaction buffer (50 mM citrate pH 4.5; 50 mM MES pH 6; 50 mM NaH2PO4/Na2HPO4 pH 7; 50 mM Tris-HCl pH 8; and 50 mM Bicine pH 9), (B) temperature, (C) ionic strength, (D) protein concentration or (E) GTP concentrations were changed. Reactions were performed for 120 min using standard conditions except the tested variable. (TIF) [file ppat.1005265.s001.tif]

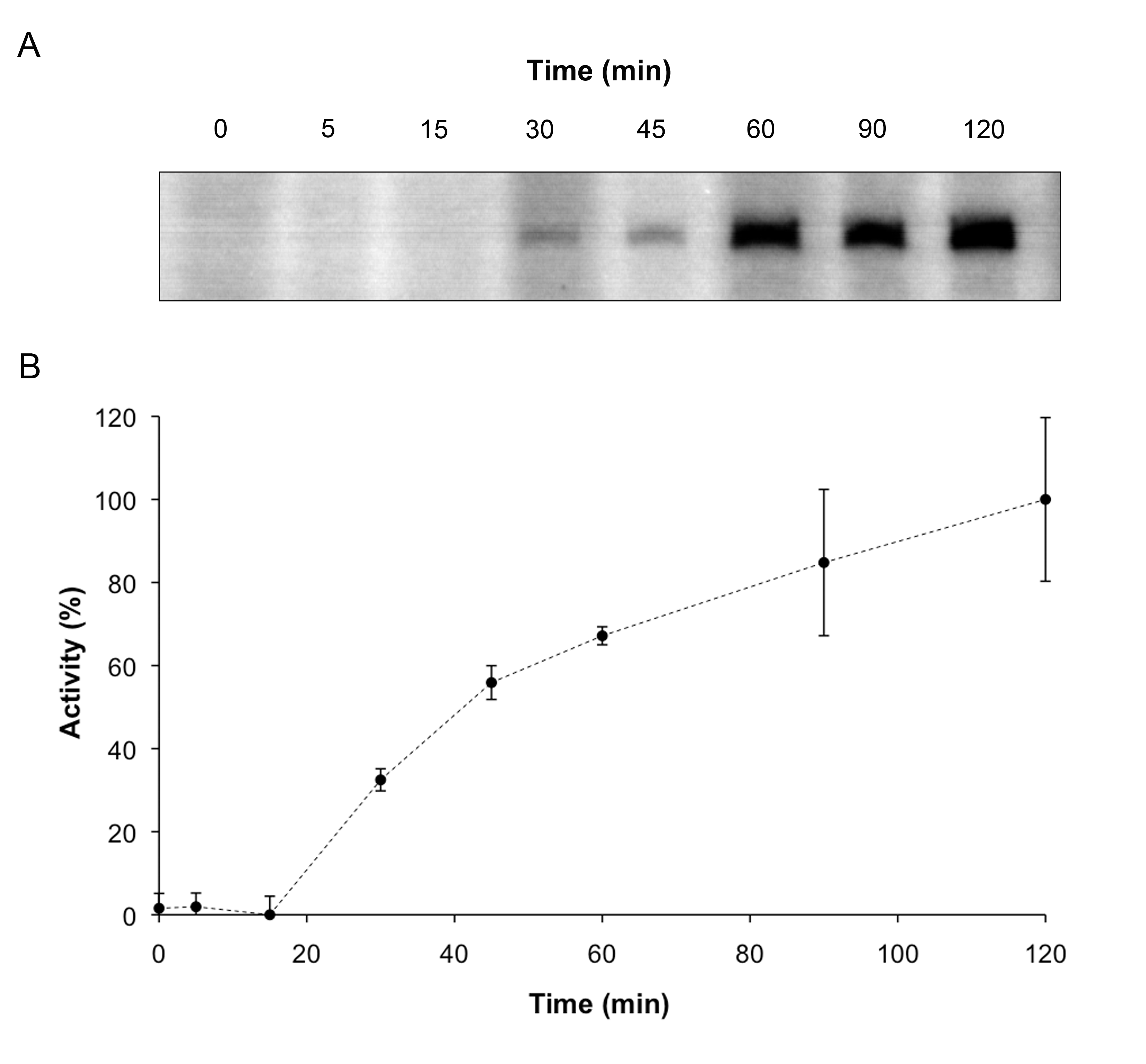

Supplement: S2 Fig — (A) Autoradiography of a 7% TBE PAGE corresponding to polymerization products of TaVpol generated under optimal reaction conditions. The reaction was stopped at different incubation times. (B) The graph corresponds to normalized liquid scintillation determination of polymerization reactions products. Each point corresponds to the average value of quantifications from three independent experiments. (TIF) [file ppat.1005265.s002.tif]

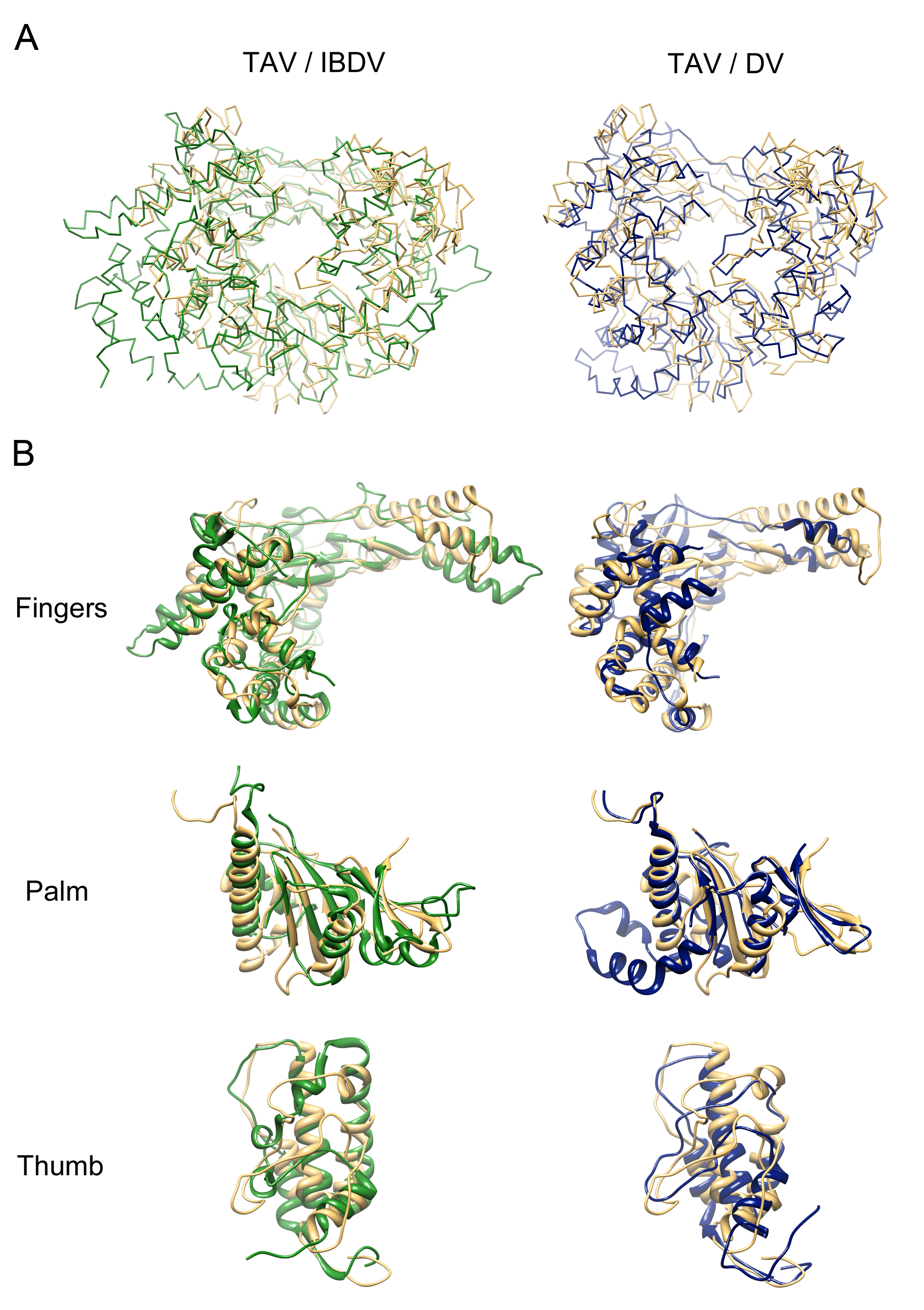

Supplement: S3 Fig — (A) Cα tracing of the TaV enzyme (gold) with the IBDV (left panel; green) and DV (right panel; dark blue) RdRP structures superimposed. (B) Ribbon diagrams of the individual, fingers (top), palm (middle) and thumb (bottom) sub-domain superimpositions. (TIF) [file ppat.1005265.s003.tif]

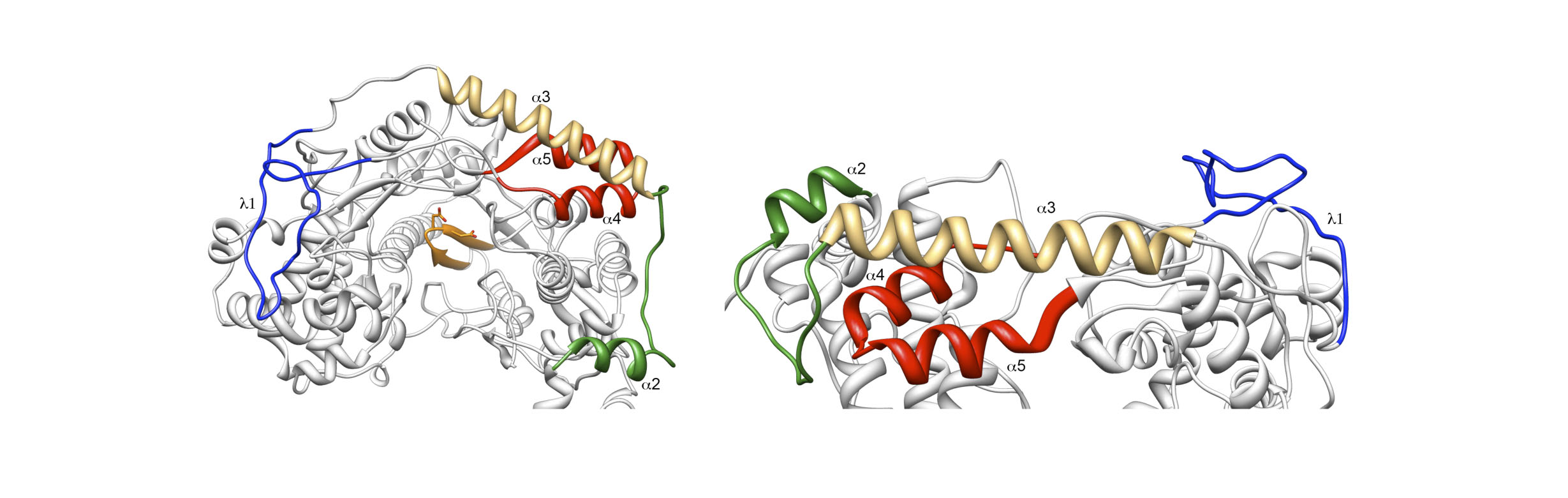

Supplement: S4 Fig — Top view of the structures closing the RdRP domain (left panel) and zoom of the back view. The interacting secondary structural elements are depicted in different colors. Extensive interactions are found between the finger helices α4 and α5 and the thumb region formed by α19, the α19-α20 loop and α21. Furthermore, the N-terminal helix α2 and the α2-α3 loop contact the thumb helices α19 and α22. This large interdomain interface results in a robust closing of the central cavity of the enzyme. (TIF) [file ppat.1005265.s004.tif]

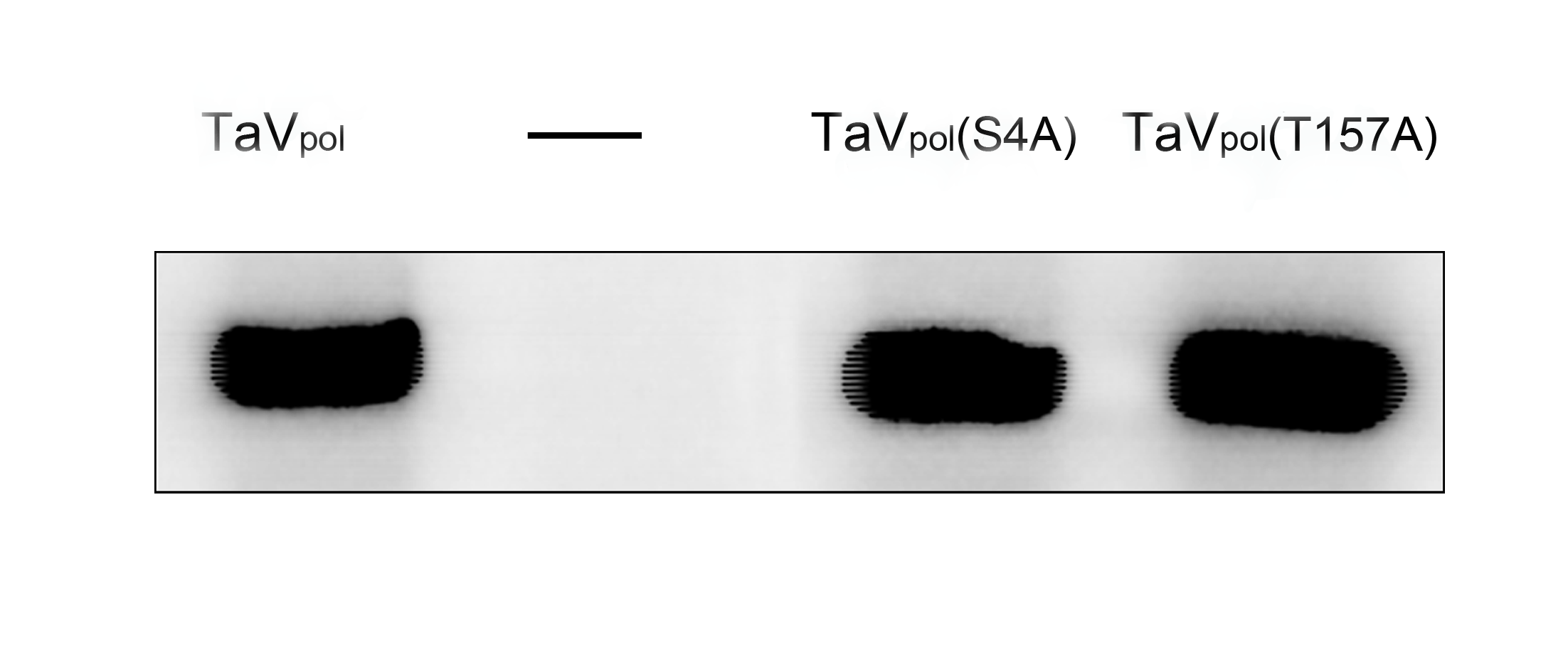

Supplement: S5 Fig — Autoradiograms of in vitro RdRP activity of TaVpol(S4A) and TaVpol(T157A) analyzed in 7% acrylamide TBE gels, showing that the polymerase mutants harboring substitutions in the predicted nucleotidylation residues, maintain the levels of RNA synthesis similar to those found in the non-mutated enzyme. (TIF) [file ppat.1005265.s005.tif]

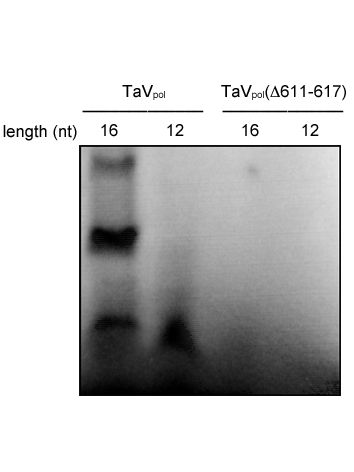

Supplement: S6 Fig — Autoradiography of the polymerization products analyzed in a 7% acrylamide TBE gel. The first and second lines correspond to the activity of TaVpol using of 16- (5’-AACCUUUUUCCACGCG) and 12-nucleotide (5’-UUUUUCCACGCG) long ssRNA templates, respectively. The third and fourth lanes correspond to the activity of TaVpol(Δ611–617) on the same 16- and 12-nucleotide templates. Reactions were performed in presence of 50 mM MES pH 6.0, 100 mM NaCl, 5 mM MgCl2, 10% glycerol, 1 mM DTT, 1 mM ATP, UTP and CTP, 0.02 mM GTP, 20 units of RNasin, and 10 μCi [α-32P] GTP, and incubated for 45 min at 35°C. (TIF) [file ppat.1005265.s006.tif]

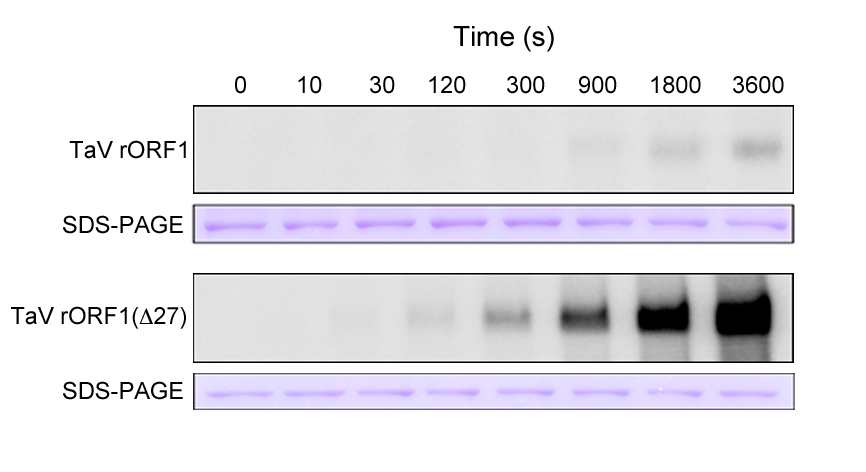

Supplement: S7 Fig — Autoradiograms of in vitro RdRP activities of full-length TaV rORF1 (top) and TaV rORF1(Δ27) (bottom). The reaction was stopped at different incubation times and the reaction products were analyzed in 7%TBE-PAGE and recorded after an exposure time of 8 h. Reactions were performed in presence of the ssRNA template of 311-nts harboring the TaV 3UTR’ sequence. The bottom image shows an 11% SDS-PAGE stained with Coomassie blue of the two proteins, used as protein loading controls. (TIF) [file ppat.1005265.s007.tif]

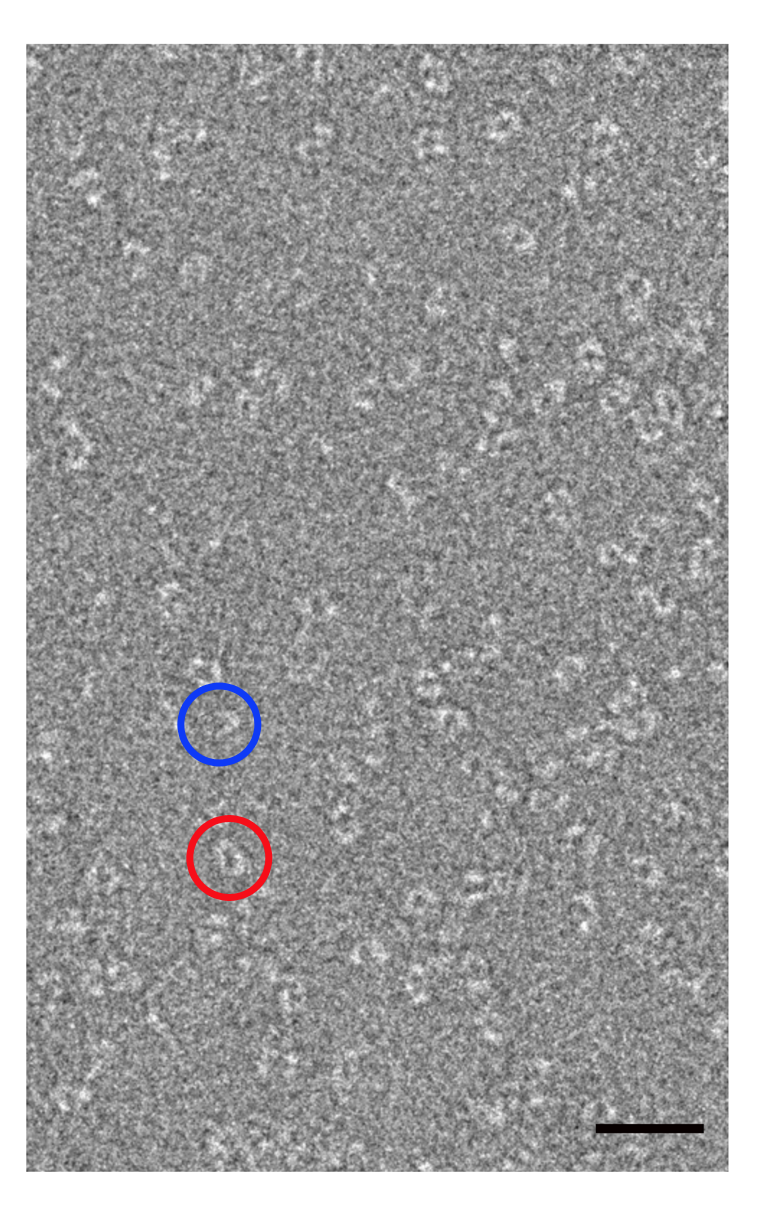

Supplement: S8 Fig — A sample of the purified enzyme was negatively stained with 2% (w/v) uranyl acetate. Red and blue circles indicate dimer and monomer respectively. Scale bar, 3 nm. (TIF) [file ppat.1005265.s008.tif]

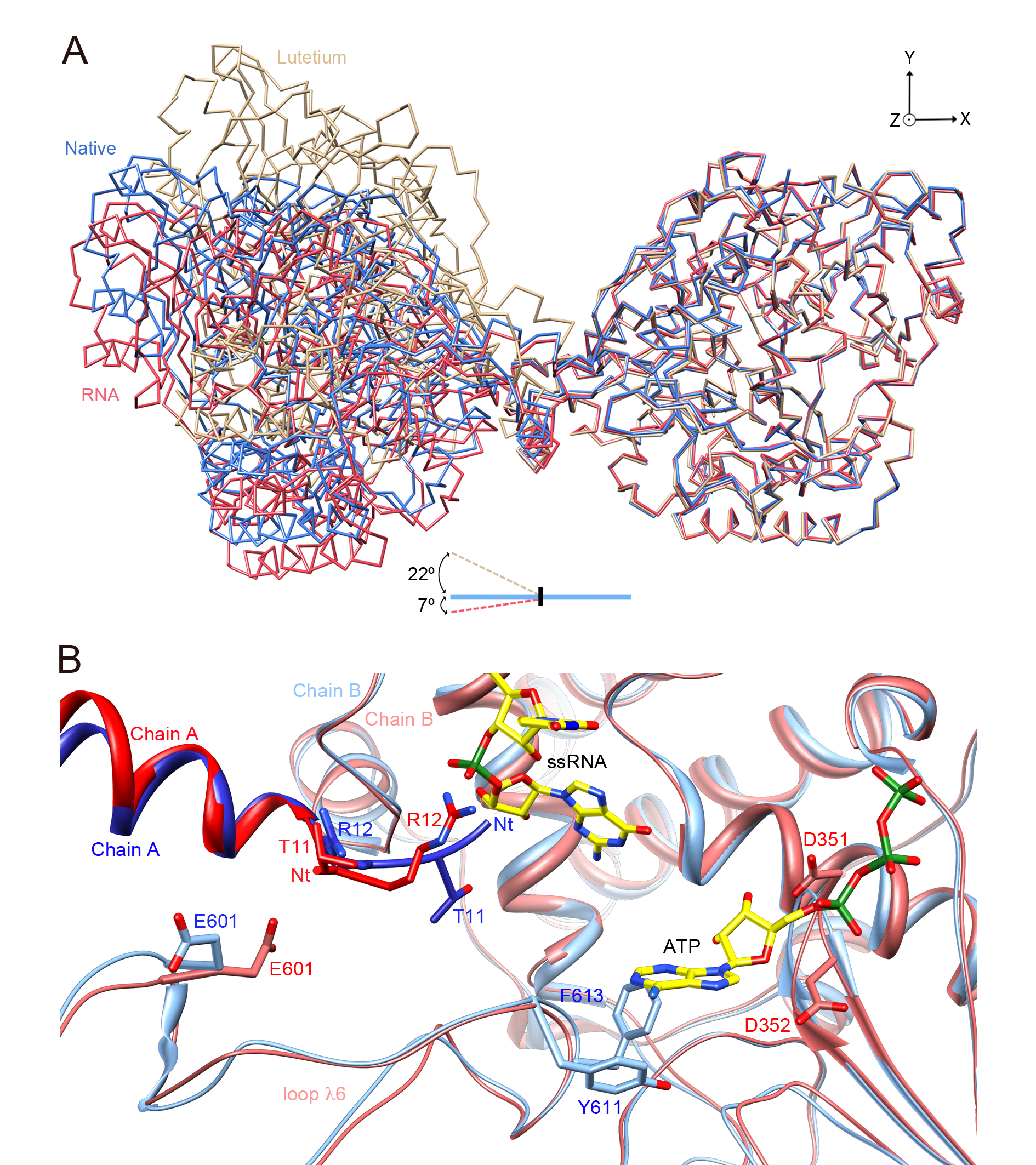

Supplement: S9 Fig — (A) The apo-form of TaVpol is shown in cyan, the RNA-ATP-bound enzyme in magenta and the Lutetium derivative in gold. One monomer of the RNA-ATP-bound structure shows a -7° rotation around the Z axes with respect to the same position of the apo-form TaVpol. The same monomer of the Lutetium structure shows a 22° and -18° rotation around Z and X axes, respectively, in comparison to the apo-form enzyme. The structural comparisons of individual monomers also show a subtle opening of the central channel. (B) Different conformations adopted by the TaVpol N-terminal ends in the apo structure (blue and cyan) and in the RNA-ATP complex (red and pink). In the TaVpol-RNA-ATP complex, the polymerase N-terminus points outwards from the central cavity leaving enough space to accommodate a ssRNA template, modeled in stick representation. (TIF) [file ppat.1005265.s009.tif]

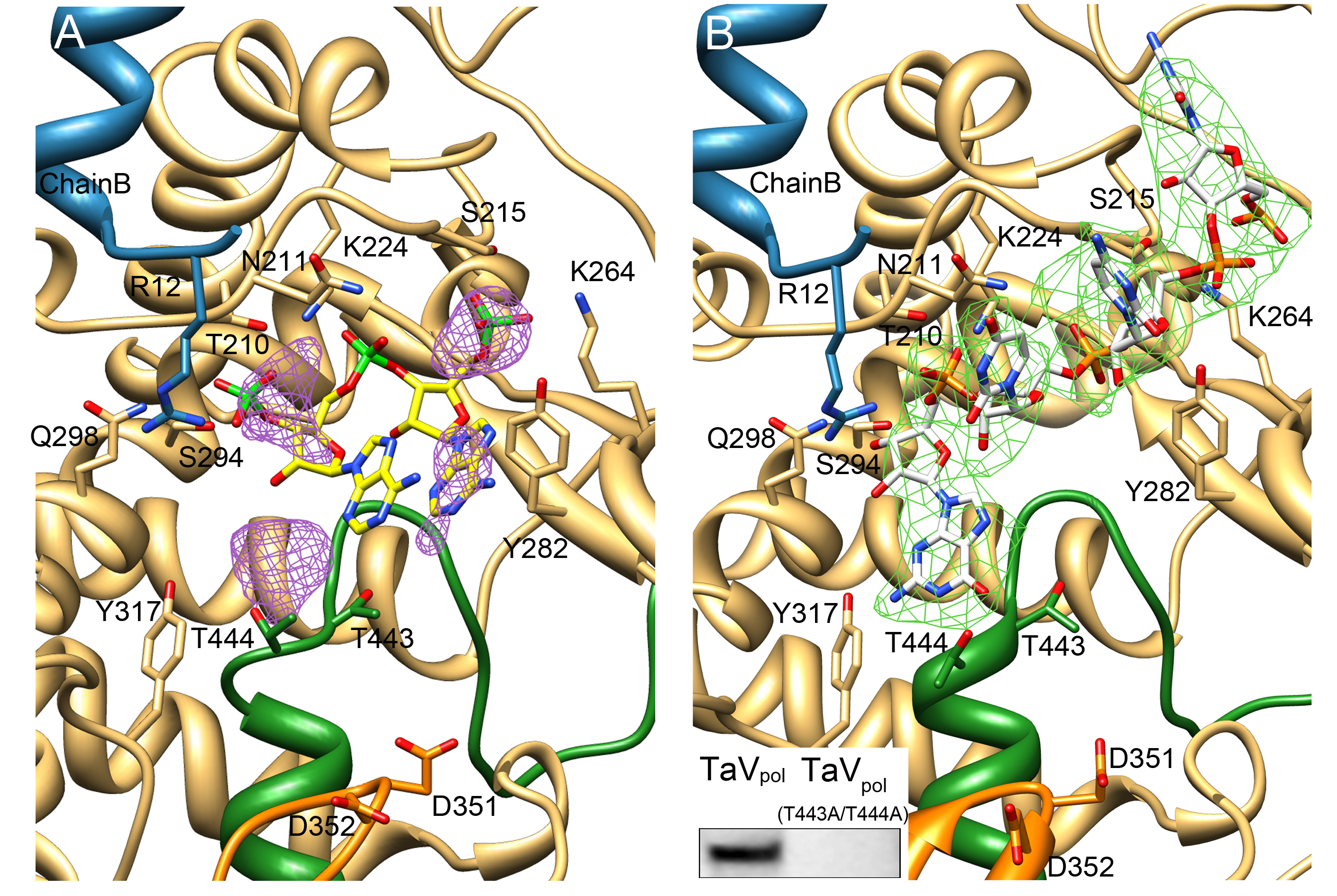

Supplement: S10 Fig — (A) View of a σA-weighted |Fo|-|Fc| electron density map (3.5σ) around the TaVpol template channel in the TaVpol-CCCAUUCGACUCCUG-ATP complex. The strong peaks of electron density (purple mesh) can be easily interpreted as the phosphate groups of a short oligonucleotide bound to the channel, in good agreement with template models derived from the superimposition of available RdRP-RNA complexes onto the TaV enzyme. The modeled template (shown as sticks in atom type color) was obtained by the superimposition of the HCV NS5B replication initiation complex (PDB ID 1WTA) onto the TaVpol active site. (B) A partial data set from other complex co-crystals, the TaVpol-GUAUACUACACCCAUUCGACUCCUG complex, have been obtained and analyzed (space group I222; a = 143.4, b = 159.0, c = 218.1 Å, with one TaVpol dimer in the crystal asymmetric unit). The X-ray data was collected using synchrotron radiation at the Swiss Light Source, PXI beam line. Unfortunately, these crystals were extremely sensitive to radiation and died before completing data collection (53.8% completeness at 3.1 Å, Rmerge = 6.8%). Attempts to merge data for different crystals failed due to the lack of isomorphism. The structure was solved by molecular replacement, using the coordinates of the unliganded TaVpol as search model. Analysis of the electron density maps revealed the presence of a partially ordered extra electron density to position a stretch of four nucleotides, most probably the 3’-end (5’-CCUG) of the template, occupying the template binding channel of the two polymerases molecules of the crystal asymmetric unit. Model refinement was performed with the program REFMAC5 [57] applying non-crystallographic symmetry restraints to the two protein molecules in the asymmetric unit. Automatic refinement was alternated with manual model rebuilding using coot [56]. The final refinement cycles converged to an Rwork of 22.3%, Rfree = 25% with good stereochemistry (r.m.s. deviations of bond lengths 0.004Å, bond angl [file ppat.1005265.s010.tif]
